# The phospholipid transporter PITPNC1 links KRAS to MYC to prevent autophagy in lung and pancreatic cancer

**DOI:** 10.1186/s12943-023-01788-w

**Published:** 2023-05-20

**Authors:** Rodrigo Entrialgo-Cadierno, Cristina Cueto-Ureña, Connor Welch, Iker Feliu, Irati Macaya, Laura Vera, Xabier Morales, Sandra Vietti Michelina, Pietro Scaparone, Ines Lopez, Elodie Darbo, Oihane Erice, Adrian Vallejo, Haritz Moreno, Ainhoa Goñi-Salaverri, David Lara-Astiaso, Nils Halberg, Ivan Cortes-Dominguez, Elizabeth Guruceaga, Chiara Ambrogio, Fernando Lecanda, Silve Vicent

**Affiliations:** 1 Program in Solid Tumours, University of Navarra, Centre of Applied Medical Research (CIMA), 55 Pio XII Avenue, 31008 Pamplona, Spain; 2 Centro de Investigación Biomédica en Red de Cáncer (CIBERONC), Madrid, Spain; 3 Imaging Unit and Cancer Imaging Laboratory, University of Navarra, CIMA, Pamplona, Spain; 4 Department of Molecular Biotechnology and Health Sciences, Molecular Biotechnology Centre, University of Torino, Turin, Italy; 5 University of Bordeaux, INSERM, BRIC, U 1312, F-33000 Bordeaux, France; 6 Molecular Therapies Program, University of Navarra, CIMA, Pamplona, Spain; 7 Wellcome - MRC Cambridge Stem Cell Institute (CSCI), Cambridge, UK; 8 Department of Biomedicine, University of Bergen, Bergen, Norway; 9 Bioinformatics Platform, University of Navarra, CIMA, Pamplona, Spain; 10 IdiSNA, Navarra Institute for Health Research, Pamplona, Spain; 11 Department of Pathology, Anatomy and Physiology, University of Navarra, Pamplona, Spain

**Keywords:** PITPNC1, KRAS, LUAD, PDAC, MYC, mTOR, Therapy

## Abstract

**Background:**

The discovery of functionally relevant KRAS effectors in lung and pancreatic ductal adenocarcinoma (LUAD and PDAC) may yield novel molecular targets or mechanisms amenable to inhibition strategies. Phospholipids availability has been appreciated as a mechanism to modulate KRAS oncogenic potential. Thus, phospholipid transporters may play a functional role in KRAS-driven oncogenesis. Here, we identified and systematically studied the phospholipid transporter PITPNC1 and its controlled network in LUAD and PDAC.

**Methods:**

Genetic modulation of KRAS expression as well as pharmacological inhibition of canonical effectors was completed. *PITPNC1* genetic depletion was performed in in vitro and in vivo LUAD and PDAC models. *PITPNC1*-deficient cells were RNA sequenced, and Gene Ontology and enrichment analyses were applied to the output data. Protein-based biochemical and subcellular localization assays were run to investigate PITPNC1-regulated pathways. A drug repurposing approach was used to predict surrogate PITPNC1 inhibitors that were tested in combination with KRASG12C inhibitors in 2D, 3D, and in vivo models.

**Results:**

*PITPNC1* was increased in human LUAD and PDAC, and associated with poor patients’ survival. PITPNC1 was regulated by KRAS through MEK1/2 and JNK1/2. Functional experiments showed PITPNC1 requirement for cell proliferation, cell cycle progression and tumour growth. Furthermore, PITPNC1 overexpression enhanced lung colonization and liver metastasis. PITPNC1 regulated a transcriptional signature which highly overlapped with that of KRAS, and controlled mTOR localization via enhanced MYC protein stability to prevent autophagy. JAK2 inhibitors were predicted as putative PITPNC1 inhibitors with antiproliferative effect and their combination with KRASG12C inhibitors elicited a substantial anti-tumour effect in LUAD and PDAC.

**Conclusions:**

Our data highlight the functional and clinical relevance of *PITPNC1* in LUAD and PDAC. Moreover, PITPNC1 constitutes a new mechanism linking KRAS to MYC, and controls a druggable transcriptional network for combinatorial treatments.

**Graphical Abstract:**

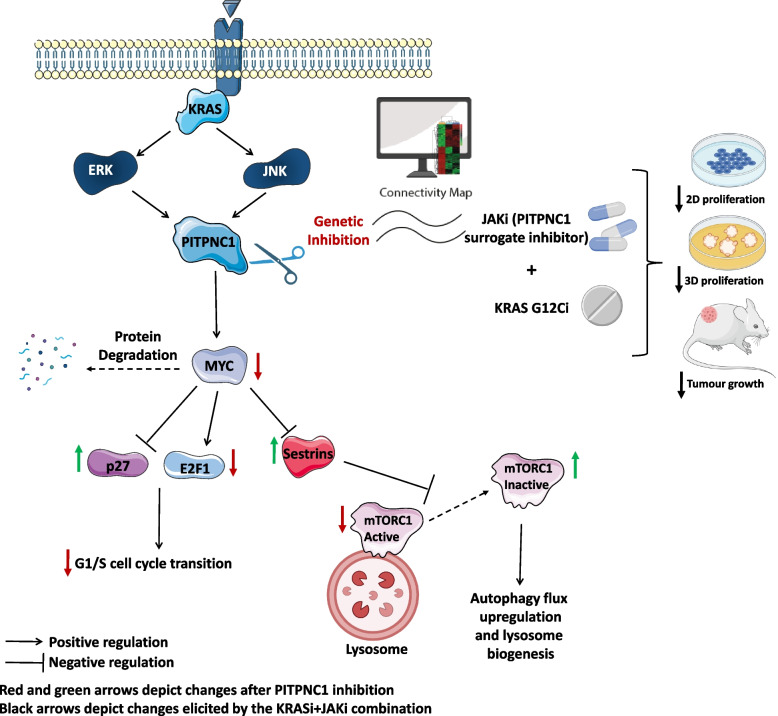

**Supplementary Information:**

The online version contains supplementary material available at 10.1186/s12943-023-01788-w.

## Background

KRAS is a driver oncogene in several epithelial tumours. In lung and pancreatic ductal adenocarcinoma (LUAD and PDAC), *KRAS* mutation frequency reaches ~ 25 and 90% of cases respectively [1, 2]. These cancers share a notable dependency on aberrant KRAS expression through activation of canonical ‘‘proximal’’ effectors, mainly the RAF-MEK-ERK and the PI3K-AKT-mTOR pathways [3, 4]. Consequently, BRAF, MEK1/2, PI3K or mTOR inhibitors were developed and progressed to clinical trials. However, such inhibitors have had limited or no impact on cancers bearing *KRAS* mutations. Activation of KRAS canonical pathways translates into transcriptomic changes that involve far many ‘‘distal’’ effectors, whose defined role in oncogenesis is less understood. The discovery of transcriptomic changes involving functional effectors in KRAS-driven cancers is thus a relevant question in the field that may lead to the identification of molecular targets for novel therapeutic strategies. Our group previously reported a KRAS signature upregulated across KRAS-driven cancers [5]. Follow up studies confirmed the relevant role of a member of the signature, the transcription factor FOSL1, in LUAD and PDAC [5] as well as in cholangiocarcinoma [6]. These data suggest that additional genes pertaining to the KRAS signature could have a functional role in mutant (mut) *KRAS* cancers.

Traditionally regarded as integral components of biological membranes, phospholipids play a relevant role as signalling elements in normal homeostasis and cancer [7]. For instance, phosphatidic acid (PA) functions as a docking site for the selective recruitment of effector proteins to local cell membrane compartments that are involved in transducing signals [8]. Also, phosphatidylinositol (PI) has a central role in the regulation of PI3K-mediated oncogenesis as a precursor to phosphatidylinositol (3,4,5)-trisphosphate (PIP3) [9]. In the KRAS setting, mut *Kras* increases the levels of phospholipids (i.e., phosphoinositide derivatives) to foster oncogenesis [10]. Conversely, depletion of phospholipid (i.e., phosphatidylserine, PS) trafficking or membrane content compromises KRAS membrane association and subsequent activation [11–13]. Hence, it is plausible that proteins regulating the distribution and availability of phospholipids may contribute to the KRAS oncogenic phenotype. A recent study describing the relevance of the PS transport proteins ORP5 and ORP8 to support KRAS oncogenic function in PDAC [14] strongly favours this idea and warrants further investigation of additional members within the phospholipid transport machinery.

The phosphatidylinositol transfer protein (PITP) family participates in phospholipid transport between cell membranes [15]. Class I PITPs, PITPα (PITPNA) and PITPβ (PITPNB) bind PI or phosphatidylcholine (PC). PITPNC1, which is classified as a Class II PITP, binds PI and PA instead of PC [16]. PITPNC1 was originally reported as a gene amplified in human breast cancer and over-expressed in breast, colon and melanoma metastasis, where it fosters the pro-metastatic phenotype via secretion of pro-invasive and pro-angiogenic mediators [17]. Subsequent studies characterizing PITPNC1 expression in human specimens revealed its association with advanced clinical stage and poor prognosis in gastric cancer [18], and with radio-resistance in rectal cancer [19]. Nonetheless, the information about PITPNC1 in cancer is far from being completed, furthermore in the context of dominant oncogenes such as *KRAS*.

In this study, we uncover PITPNC1 as a KRAS-dependent gene with functional implications in LUAD and PDAC, in part by the unanticipated regulation of autophagy by modulating mTOR localization via cMYC (hereinafter referred to as MYC). Most importantly, PITPNC1 controls a druggable transcriptome that offers opportunities for therapeutic intervention in both cancers.

## Materials and methods

### Cell lines

Human LUAD cell lines (wild-type -wt- *KRAS*: H2126, H1568; mut *KRAS*: A549, H23, H2009, H1792, H358, H2347), PDAC cell lines (mut *KRAS*: PATU8902, HPAFII, PANC1, MiaPaca2) or normal human pancreatic duct epithelial (HPDE) cells, H6C7, were used. LUAD and PDAC cells were grown in fully supplemented RPMI1640 or DMEM media (Gibco) respectively. H6C7 were grown in serum-free Keratinocyte medium (Gibco). Cell lines were authenticated by the Genomics Unit at CIMA, using Short Tandem Repeat profiling (AmpFLSTR® Identifiler® Plus PCR Amplification Kit). *Hras*^−/−^; *Nras*^−/−^; *Kras*^lox/lox^; RERTn^ert/ert^ (hereinafter referred to as *Kras*^lox/lox^) mouse embryo fibroblasts (MEFs) were grown in fully supplemented DMEM (Gibco) and have been described previously [20]. Mycoplasma test was performed in all cell lines every other week using the MycoAlert Mycoplasma Detection Kit (LONZA). Only mycoplasma-free cell lines were used.

### Reagents

Specific shRNAs oligonucleotides against *PITPNC1* (sh6: TRCN0000059479, sh7: TRCN0000059481), *MYC* (sh42 TRCN0000039642, sh89 TRCN0000010389) were annealed and cloned into a pLKO.1 lentiviral vector (Addgene #10,878). The tet-pLKO-shKRAS (TRCN0000033260) and shRNA against green fluorescence protein (GFP) were already published [5]. For CRISPR knockout experiments, specific sgRNAs against *LKB1* (GTACTCCATCACCATATACG and CTTCAAGGTGGACATCTGGT), *Lkb1* (GACACTCAAGATCTCCGACCT), Keap1 (GGGTTCGGTTACCGTCCTGCG), and *Trp53* (GAACAGATCGTCCATGCAGTG) were annealed and cloned into lentiCRISPRv2 (Addgene #52,961) or lentiCRISPRv2-mCherry (Addgene #99,154) plasmids. Wt (4B) and mut (G12D, G12C and G12V) *KRAS* cDNAs were from the RAS Initiative (Addgene, Kit #1,000,000,089) and were described previously [5]. The pBABE-puromycin PITPNC1-flagged plasmid was described earlier [17]. For MEFs experiments, *KRASG12C*, *KRASG12D*, *KRASG12V*, *KRASG12R*, *KRASG12S*, *KRASG13D* and *KRASQ61H* retroviral plasmids were created by point mutagenesis from pBABE HA-tagged *KRAS*WT plasmid (provided by Channing Der, Addgene plasmid # 75,282). A MYC cDNA in a pDONR221 vector was provided by Alejandro Sweet-Cordero (University of California San Francisco, USA) and cloned into a pLenti6/V5-DEST using the Gateway system (Thermofisher).

### Virus production and infection

*KRAS*MUT-expressing retroviruses were generated by co-transfection of pBABE plasmids together with pAmpho plasmid into HEK293T cells using FuGENE HD Transfection Reagent (Promega). The retroviruses were transduced into *Kras*^lox/lox^ MEFs followed by 2 weeks of puromycin selection (1 μg/mL) in complete DMEM medium. To obtain *Kras* null-*KRAS*MUT clones, cells were then cultured in the presence of 4-hydroxytamoxifen (4OHT) (Sigma, 600 nM) for another 2 weeks in order to achieve complete deletion of endogenous *Kras* allele. The remaining retrovirus and lentivirus were produced as previously described [21], then filtered and applied directly to cells for infection at a MOI lower than 1. Selection was done with puromycin, neomycin, or blasticidin (Sigma).

### Quantitative PCR (qPCR) analysis

mRNA analysis was done as published earlier [6]. Primers sequences are listed as Suppl. Material.

### Immunoblotting

Immunoblot analysis was performed as previously described [5]. Antibody information is found as Suppl. Material.

### Cell proliferation assay

Cell proliferation assays were done as described earlier [6]. A detailed explanation is found as Suppl. Material.

### Clonogenic assay

Clonogenic assays were performed as previously described [6]. A detailed explanation is found as Suppl. Material.

### Drug combination studies in vitro

Cell lines were plated at a density ranging from 300 to 2,000 cells in 96-well plates, treated on the following day with single drugs or combination, and cultured for 5 days. At day 5, cells were fixed with 4% formaldehyde (Panreac) for 15 min at RT, stained with crystal violet solution (Sigma-Aldrich) (1% crystal violet in H_2_O) for 15 min. Relative growth was quantified by measuring absorbance at 570 nm after crystal violet dissolution with 10% acetic acid. SynergyFinder software (https://synergyfinder.fimm.fi/) was used to determine the potential synergism of two drugs. Bliss score values > 10 were considered synergistic.

### Long-term drug combination assays

Long-term drug combination assays were performed and analysed as previously described [6]. A detailed explanation is found as Suppl. Material.

### 3D culture assays

3D culture assays were done as described previously [22]. A detailed explanation is found as Suppl. Material.

### Cell cycle and apoptosis assays

Cell cycle and apoptosis assays were performed as previously described [6]. A detailed explanation is found as Suppl. Material.

### Drug repurposing

The Connectivity Map (https://clue.io/) was used to predict genes or pharmacological compounds able to phenocopy a PITPNC1-knockdown signature induced by two specific shRNAs.

### Pharmacological inhibitors

SP600125 (JNKi) and hydro-chloroquine (C6628) were from Sigma; BIX02189 (MEK5i) was from Tocris; Trametinib (MEKi), GSK2126458 (PI3Ki), Fedratinib (JAK2i), BI2536 (PLK1i), MG-132 and Sotorasib (KRASG12Ci) were from MedChemExpress.

### Animal work

All experiments in mice were performed according to the institutional Animal Care Committee (CEEA) of the University of Navarra under the protocols CEEA #057–18 approved by the regional Government of Navarra. A detailed description of the mouse experiments is provided as Suppl. Material.

### Microscope image acquisition

Human tissue slides were scanned at 40 × magnification and digitalized using the Aperio Scan-Scope XT Slide Scanner (Aperio Technologies). Mouse slides were scanned at 40 × magnification and acquired with Aperio CS2 Leica Biosystems.

### Immunofluorescence

Immunofluorescence to determine mTOR localization was done using anti-mTOR (#2983 7C10 CST) and AlexaFluor 488-conjugated anti-LAMP1 antibody (clone H4A3, 328,609 Biolegend). A detailed explanation of the protocol and analysis is provided as Suppl. Material.

### RNA sequencing (RNAseq) and analysis

Low full-length RNA seq libraries were prepared by adapting the Smart-seq 2 protocol to 1 ng of RNA [23]. RNA sequencing data analysis was performed using the following workflow: (1) the quality of the samples was verified using FastQC software (https://www.bioinformatics.babraham.ac.uk/projects/fastqc/); (2) the alignment of reads to the human genome (hg38) was performed using STAR [24]; (3) gene expression quantification using read counts of exonic gene regions was carried out with featureCounts [25]; (4) the gene annotation reference was Gencode v35 [26]; and (5) differential expression statistical analysis was performed using R/Bioconductor [27]. Data are publicly available in GEO database with the accession number GSE205767. A detailed description of the RNA-seq analysis is provided as Suppl. Material.

### CNV analysis

Copy number variation data from the The Cancer Genome Atlas (TCGA) LUAD dataset [28] were downloaded and analyzed with GISTIC2 [29].

### LOH analysis

The association between loss of heterozygosity at the KRAS locus and PITPNC1 gene expression in mut *KRAS* LUAD was assessed in R using the ploidy estimated by ABSOLUTE and RNAseq batch corrected matrix published in Hoadley et al [30]. The statistical significance was measured by a Wilcoxon’s test.

### PITPNC1 expression profile

The expression profile of PITPNC1 gene was studied in the TCGA LUAD transcriptome dataset (downloaded from https://tcga-data.nci.nih.gov/tcga/tcgaHome2.jsp) to assess differential expression in patients. Data processing and statistical analyses were performed as previously described [5].

### Survival analyses

Survival analyses were conducted on both *PITPNC1* gene and a *PITPNC1* gene set in the TCGA [28] and Shedden et al [31] data sets and in the International Cancer Genome Consortium (ICGC) [32] and TCGA PDAC data sets. Log-rank test was used to calculate differences in Kaplan–Meier curves [33]. For gene set studies, a summation of all the genes for a particular sample was calculated as previously described [34]. Survival analyses were done with R [27] and *p*-values < 0.05 were statistically significant.

### PITPNC1 gene set enrichment analyses

GSEA analyses were performed using the dPITPNC1 gene set. Enrichment was studied in samples with inhibition of KRAS expression (GSE196596 and GSE103021), in LUAD patients with *KRAS* mutation (GSE36133, GSE12667, GSE31210, and GSE26939), in PDAC patients (GSE15471 and GSE16515) or in PDAC models (GSE32277). Data processing of each experiment was previously described [5]. FDR < 0.05 was considered statistically significant and the results were represented using GSEA Multi-sample Running Enrichment plots (https://github.com/GryderArt/VisualizeRNAseq).

### Statistics

Sample size was chosen using www.biomath.info/power/ttest.htm or based on similar experiments previously published by the authors. For comparison of two groups, normality (Shapiro–Wilk test) and variance (Levene test) was assessed. Groups with normal distribution followed a t-test. Non-normal samples were analysed using a Mann–Whitney test (equal variances) or a Median test (unequal variances). For comparison of more than two groups, a residual test was performed to study normality and Levene test assessed homoscedasticity. ANOVA, Brown Forsythe, Kruskal Wallis or Median tests were performed depending on data distribution. A post-hoc test (Dunnet or Bonferroni) explored paired comparisons. All analyses were two-tailed. Error bars correspond to either standard deviation (S.D., *n* < 8) or standard error of the mean (S.E.M, n ≥ 8). Survival analyses were done using the Log-rank test. Statistical analyses were done with GraphPad software v8.

## Results

### PITPNC1 is regulated by *KRAS* oncogene and predicts poor survival in LUAD and PDAC

**Fig. 1:**
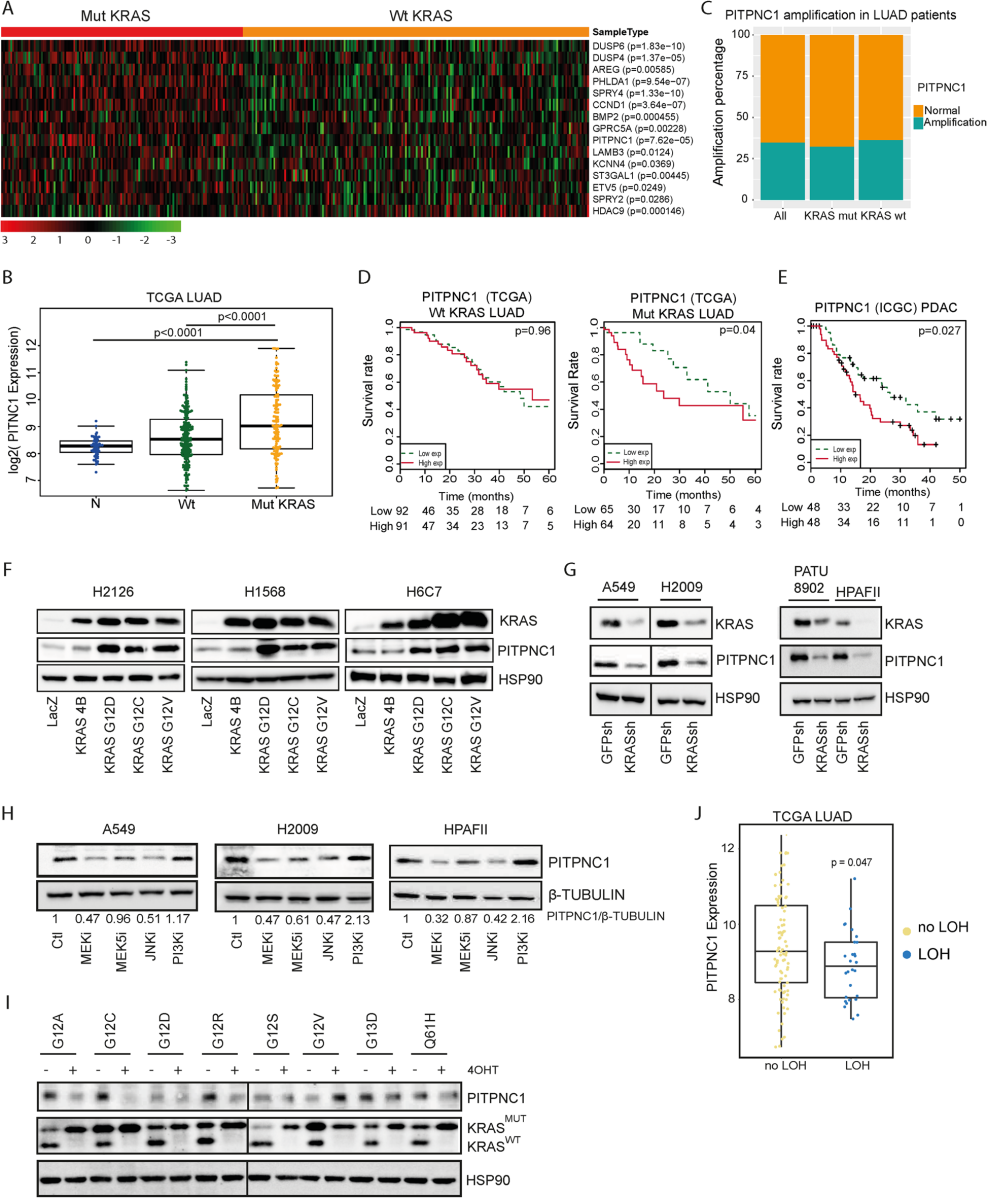
PITPNC1 is upregulated in *KRAS*-mutated LUAD and PDAC and predicts poor survival. **A** Heatmap of upregulated genes in The Cancer Genome Atlas (TCGA) LUAD data set comparing expression profiles of wt and mut *KRAS* LUAD patients. **B ***PITPNC1* mRNA expression levels in normal lung (N), wild type (wt) and mutant (mut) *KRAS* LUAD. Mut vs wt *KRAS* (*p* < 0.0001) or vs N (*p* < 0, 0001). **C** *PITPNC1* gene amplification percentage (GISTIC2 analysis) in mut and wt *KRAS* LUAD samples, or both (*p* = 0.815). **D** Kaplan–Meier survival analysis of LUAD patients, stratified based on *KRAS* status and *PITPNC1* expression. Data from TCGA database: wt *KRAS* (Log-rank test *p* = 0.96) and mut *KRAS* (Log-rank test *p* = 0.04). **E** Kaplan–Meier survival analysis of PDAC patients stratified by *PITPNC1* expression. Data from ICGC database (Log-rank test *p* = 0.027). **F** Western blot of PITPNC1 and KRAS expression in H2126 and H6C7 cells, expressing a control (LacZ) or overexpressing KRAS (wt *KRAS4B* or mut *KRASG12D*, *G12C* or *G12V*). Twenty μg of protein were loaded per sample. HSP90 and β-TUBULIN were used as loading markers. **G** Western blot of PITPNC1 and KRAS expression in A549, H2009, PATU8902 and HPAFII cells, expressing a control (GFPsh) or an inducible *KRAS* shRNA (KRASsh) (activated by 1 µg/ml doxycycline). Twenty μg of protein were loaded per sample. HSP90 were used as loading markers. **H** Western blot of PITPNC1 expression in A549, H2009 and HPAFII cells treated for 24 h with pharmacologic inhibitors: trametinib (MEKi, 0.5 μmol/L), BIX02189 (MEK5i, 10 μmol/L), SP600125 (JNKi, 10 μmol/L) or GSK2126458 (PI3Ki, 0.1 μmol/L). Twenty μg of protein were loaded per sample. β-TUBULIN was used as loading marker. **I** Western blot of PITPNC1 and KRAS expression in *Kras*^lox/lox^ MEFs transduced with different human HA-tagged *KRAS* mutants (G12C, G12D, G12V, G12R, G12S, G13D and Q61H). 4OHT: 600 nM. **J ***PITPNC1* mRNA expression levels in no loss of heterozygosity (no LOH) and loss of heterozygosity (LOH) TCGA LUAD patients. no LOH vs LOH (*p* = 0.047)

To identify novel effectors with relevance to KRAS oncogenesis, we used a gene signature derived from experimental models expressing mut *KRAS* (*n* = 41 genes) reported by our group [35]. We queried this gene signature against TCGA data set by comparing gene expression profiles of wt and mut *KRAS* LUAD patients. Among the 15 differentially expressed genes, *SPRY4*, *DUSP6*, *CCND1*, *PHLDA1*, *DUSP4*, and *PITPNC1* were the most robustly upregulated in *KRAS*-mutated patients (*p* < 0.0001) (Fig. 1A). We focused our attention on the phosphatidylinositol cytoplasmic transfer protein 1 (PITPNC1) which, unlike the other genes, had not been previously linked to *KRAS* oncogene biology. *PITPNC1* was also upregulated when compared to normal lung tissue, indirectly suggesting a link to the tumour phenotype (Fig. 1B). *PITPNC1* mRNA increase was dependent on *KRAS* oncogene expression, since the presence of other dominant oncogenic drivers (e.g. *BRAF* or *EGFR*) did not affect *PITPNC1* transcript levels (Suppl. Figure 1A and B). *PITPNC1* upregulation was not due to differential *PITPNC1* amplification in mut vs wt *KRAS* LUAD patients either (*p* = 0.815) (Fig. 1C). Thus, the differential transcriptional regulation of *PITPNC1* may be a consequence of aberrant KRAS activation. We further tested PITPNC1’s clinical role in human cancer by performing survival analysis in LUAD patients. High *PITPNC1* expression was associated with poor overall survival in mut *KRAS* patients but not in wt (Fig. 1D). Since *PITPNC1* was part of a mut *KRAS* signature that included genes with a role in LUAD and PDAC, we studied human PDAC specimens. Notably, high *PITPNC1* was also a worse prognosis marker in PDAC (Fig. 1E).

The clinical data led us to test the connection between *KRAS* and *PITPNC1* via genetic gain- and loss-of-function experiments in lung and pancreas cellular models. Overexpression of mut *KRAS* (G12D, G12C and G12V) in wt *KRAS* LUAD cells (H2126 and H1568) and in immortalized normal human pancreatic duct epithelial cells (H6C7) increased PITPNC1 protein and mRNA levels (Fig. 1F). Such PITPNC1 upregulation was also observed in human LUAD patients with different *KRAS* mutations (Suppl. Figure 1C). Conversely, *KRAS* inhibition in LUAD (A549, H2009, H1792) and PDAC (PATU8902, HPAFII) cells using a specific shRNA decreased PITPNC1 protein (Fig. 1G, and Suppl. Figure 1D and E). PITPNC1 was consistently downregulated upon inactivation of MEK1/2 and JNK1/2 in both LUAD (A549, H2009) and PDAC (HPAFII) cells (Fig. 1H and Suppl. Figure 1F), indicating a regulation by KRAS through different effector pathways. Notably, PITPNC1 was the unique member of the PITP family controlled by KRAS, as the expression of *PITPNA*, *PITPNB*, *PITPNM1*, *PITPMN2* and *PITPNM3* did not change upon KRAS genetic modulation (Suppl. Figure 1G-I).

In addition to KRAS activating mutations [36], an imbalance between wt and mut *KRAS* alleles can influence cancer cells’ fitness, expression profile and therapy response in LUAD and PDAC [37–40]. Thus, we investigated PITPNC1 levels in relationship to KRAS dosage. First, we used *Kras*^lox/lox^ MEFs expressing different *KRAS* mutations to study PITPNC1 in the context of loss-of-heterozygosity (LOH) [39]. Similar to human cell lines, exogenous expression of the various *KRAS* mutations increased PITPNC1 expression (Suppl. Figure 1J). Notably, Cre-excision of the wt allele in MEFs via 4-OHT treatment reduced PITPNC1 levels in all mutants but G12V (Fig. 1I). Such decrease was also observed in mut *KRAS* human samples of the LUAD TCGA data set (*p* = 0.047) (Fig. 1J). Second, we assessed the impact of mut *KRAS* amplification on *PITPNC1* expression in the LUAD data set. However, no significant differences were found (Suppl. Figure 1K). These data may indicate that PITPNC1 represents a functional node downstream of KRAS integrating signals from receptor tyrosine kinases which become activated upon mut *KRAS* expression and require wt *KRAS* for downstream signalling.

Given the relevance of concurrent mutations in mut *KRAS* LUAD prognosis and response to therapy [41], we explored the association of *PITPNC1* expression with prevalently mutated tumour suppressor genes (TSGs). *LKB1* mutations were mostly found in mut *KRAS* with high *PITPNC1* expression (*p* = 0.005) while *ARID1A* mutations appeared mostly in low *PITPNC1*-expressing tumours (*p* = 0.0208) (Suppl. Figure 2A). These results led us to test the impact of *LKB1* mutations on PITPNC1 expression. CRISPR/Cas9-based *LKB1* knockout in *KRAS*-mutated LUAD cells (H2009) enhanced PITPNC1 expression (Suppl. Figure 2B). This finding was further recapitulated in mouse LUAD cell lines driven by mut *Kras* (KLA and LKR10) upon *LKB1* abrogation with specific sgRNAs (Suppl. Figure 2C). Thus, PITPNC1 is regulated by KRAS through MEK1/2 and JNK1/2 signalling pathways, and its expression may be exacerbated by *LKB1* loss.

### PITPNC1 inhibition reduces cell proliferation in vitro and impairs tumour growth in vivo in LUAD and PDAC

**Fig. 2:**
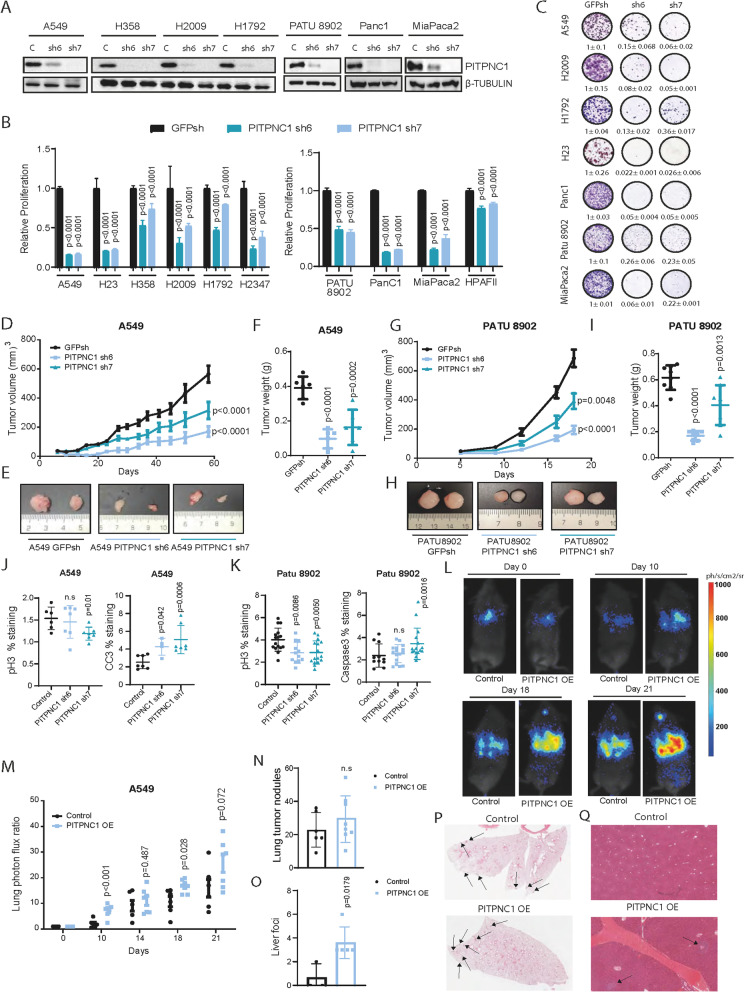
PITPNC1 inhibition in LUAD and PDAC cells reduce cell proliferation and impair tumour growth in vivo. **A** Western blot of PITPNC1 expression in A549, H358, H2009, H1792 LUAD cell lines and PATU8902, Panc1, MiaPaca2 PDAC cell lines transfected with a control (GFPsh) or a specific shRNA against *PITPNC1* (*PITPNC1* sh6 and sh7). Twenty μg of protein were loaded per sample. β-TUBULIN was used as loading marker. **B** Relative proliferation of A549 H23, H358, H2009, H1792, H2347 LUAD cell lines and PATU8902, Panc1, MiaPaca2 and HPAFII PDAC cell lines. Cells were transfected with a control (GFPsh) or a specific shRNA against *PITPNC1* (*PITPNC1* sh6 and sh7) (Dunnett´s multiple comparation test). **C** Representative images and quantification of clonogenic ability (mean ± std. error). **D** Tumour volume (mm^3^) of A549-derived xenografts (*n* = 6) (Dunnett’s multiple comparison test). **E** Representative images of tumours of D. **F** Tumour weight (g) of A549-derived xenografts (*n* = 6) of D at end point. **G** Tumour volume (mm^3^) of PATU8902-derived xenografts (*n* = 8) (Dunnett’s multiple comparison test). **H** Representative images of tumours of G. **I** Tumour weight (g) of PATU8902-derived xenografts (*n* = 8) of G at end point. **J** pH3 and CC3 quantification of A549-derived xenografts of D at end point. (Mann Whitney test). **K** pH3 and CC3 quantification of PATU8902-derived xenografts of G at end point (Mann Whitney test). **L** Representative images of lung photon flux ratio of A549 GFP/luciferase *PITPNC1*-overexpressing cells OE compared with the control (GFP/luciferase) (*n* = 8) at the indicated days. **M** Lung photon flux ratio of L (Bonferroni´s multiple comparison test). **N** Lung tumour nodules quantification on the lungs extracted from L (Mann Whitney test). **O** Liver foci quantification in the liver extracted from L (Mann Whitney test). **P** Representative images of lung tumour nodules quantification from N. **Q** Representative images of liver foci quantification from O

To characterise the functional role of PITPNC1, genetic depletion using two independent shRNAs, one of which had been previously validated via rescue experiments [17], was carried out in a panel of LUAD (*n* = 6) and PDAC (*n* = 4) cell lines (Fig. 2A). *PITPNC1* inhibition consistently reduced cell proliferation of all cell lines (Fig. 2B). Likewise, a decreased colony-forming capacity was also observed in both tumour types (Fig. 2C). However, we did not find a consistent effect on apoptosis in *PITPNC1*-depleted cells (Suppl. Figure 3A and B).

Next, we investigated if PITPNC1 is necessary for KRAS-driven tumourigenesis in vivo. First, LUAD cell lines infected with *PITPNC1* shRNAs were subcutaneously injected in immunocompromised mice. *PITPNC1* knocked-down cells generated tumours of a smaller volume and weight than controls (Fig. 2D-F). *PITPNC1* abrogation in PDAC cells also impaired tumour growth, yielding lighter tumours (Fig. 2G-I). The effect of *PITPNC1* loss in vivo was related to decreased tumour proliferation and enhanced cytotoxic activity in these models (Fig. 2J and K, and Suppl. Figure 3C).

Complementary to *PITPNC1* inhibition experiments, the effect of its overexpression was also assessed in mut *KRAS* LUAD cell lines (A549 and H358) (Suppl. Figure 3D). No effect on colony formation was observed (Suppl. Figure 3E). Moreover, exogenous *PITPNC1* did not confer a growth advantage in vivo when cells were injected subcutaneously in immunodeficient mice (Suppl. Figure 3F-K).

Mut *KRAS* LUAD harbouring inactivating *LKB1* mutations display poor prognosis, in part due to an enhanced metastatic potential [41, 42]. Since increased PITPNC1 expression was observed upon *LKB1* loss, we explored *PITPNC1* overexpression in the metastatic setting. A549 cells were first constructed to express luciferase and transduced with a *PITPNC1*-expressing or a control vector. We used a mouse model of lung colonization in vivo where cancer cells initially seed in the lungs after intravenous injection (~ 10–15 min post-injection) (Fig. 2L). Subsequent bioluminescence monitoring revealed that, on week 1, *PITPNC1*-expressing cells colonized the lung more efficiently. Interestingly, while the bioluminescence signal of the two groups become closer by week 2, it increased in cancer cells over-expressing *PITPNC1* at week 3 and 4 (Fig. 2M). Macroscopic and microscopic analysis of tissues at endpoint revealed a higher number of metastatic liver foci in the group of mice injected with *PITPNC1*-overexpressing cells while the lung tumor burden was similar (Fig. 2N-Q), suggesting that secondary metastasis to the liver contribute to the distinct bioluminescence signal. No differences in the migratory capacity in vitro or the metastatic tropism in vivo of control and *PITPNC1*-overexpressing cells were detected that could explain these findings (Suppl. Figure 3L-N), suggesting the involvement of heterotypic interactions as described in breast cancer [17]. Thus, PITPNC1 upregulation contributes to the metastatic phenotype of mut *KRAS* LUAD.

### A *PITPNC1* gene signature features KRAS-regulated genes and predicts poor survival in LUAD and PDAC

**Fig. 3:**
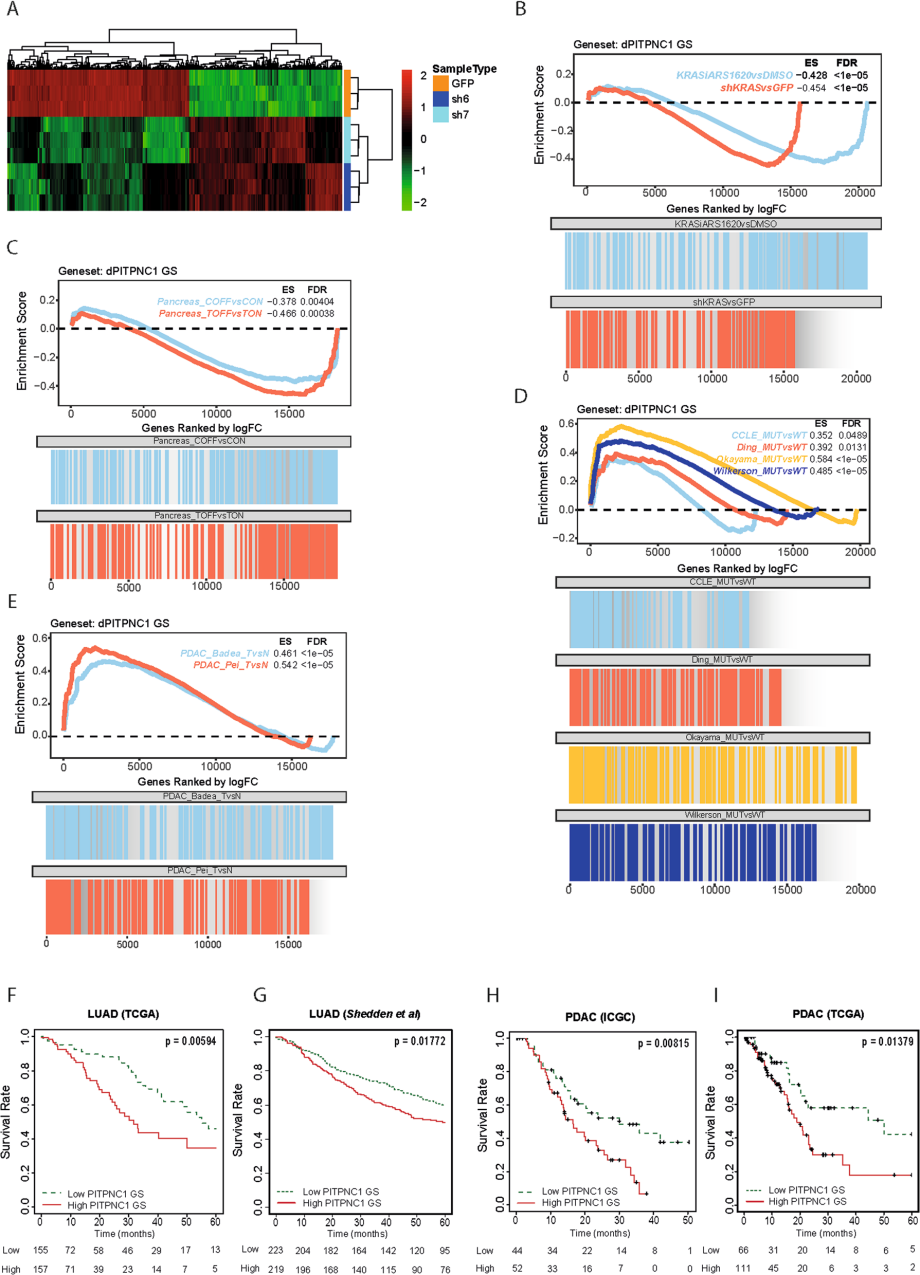
A PITPNC1 gene signature features KRAS-regulated genes and predicts poor LUAD and PDAC patients’ outcome. **A** Heat map of downregulated and upregulated genes in A549 cells after *PITPNC1* inhibition with two specific shRNAs (sh6 and sh7) or control (GFPsh). **B** Gene set enrichment analysis (GSEA) of the dPITPNC1 gene signature in the comparison of both genetically and pharmacologically KRAS inhibition (tet-shKRAS, activated by 1 µg/ml doxycycline, or KRASiARS1620 respectively) vs control (GFP or DMSO respectively). **C** GSEA of the dPITPNC1 gene signature in the comparison of gene expression data from cancer cell lines (iKrasC) and xenograft tumours (iKrasT) derived from an inducible genetically engineered mouse (GEM) model of Kras-driven PDAC in which doxycycline administration activates expression of a mutant *Kras* allele. **D** GSEA of the dPITPNC1 gene signature in the comparison of mut vs wt *KRAS* LUAD in four data sets. **E** GSEA of the dPITPNC1 gene signature in the comparison of PDAC vs normal tissue in two data sets. **F** Survival analysis of LUAD patients (TCGA data set) stratified by the dPITPNC1 gene signature (Log-rank test *p* = 0.0059). **G** Survival analysis of LUAD patients (*Shedden *et al. data set) stratified by the dPITPNC1 gene signature (Log-rank test *p* = 0.01772). **H** Survival analysis of PDAC patients (ICGC data set) stratified by the dPITPNC1 gene signature (Log-rank test *p* = 0.0081). **I** Survival analysis of PDAC patients (TCGA data set) stratified by the dPITPNC1 gene signature (Log-rank test *p* = 0.0137)

To get a better understanding of PITPNC1 as a KRAS effector, we interrogated the transcriptome of *KRAS*-mutated LUAD cells (A549) after *PITPNC1* inhibition with two shRNAs. A total of 429 genes were found differentially expressed (logFC ± 1, B > 0) with regard to control cells (Fig. 3A). The downregulated *PITPNC1* gene signature (dPITPNC1 GS; *n* = 233 genes), which a priori would contain transcriptional targets whose overexpression fosters the oncogenic phenotype, was used. This signature was queried against two independent data sets where genetic or pharmacological blockade of KRAS, via a tet-inducible *KRAS* shRNA or the KRASG12C ARS160 inhibitor respectively, was carried out. A consistent enrichment of the dPITPNC1 GS in genes repressed upon KRAS inhibition was found (Fig. 3B). To expand these findings to the pancreas setting, we took advantage of gene expression data from cancer cell lines (iKrasC) and xenograft tumours (iKrasT) derived from an inducible genetically engineered mouse (GEM) model of Kras-driven PDAC in which doxycycline administration activates expression of a mut *Kras* allele [43] (Fig. 3C). In both data sets, a large overlap of the dPITPNC1 GS with genes decreased after oncogenic *KRAS* inactivation was found, suggesting that multiple PITPNC1-regulated genes are part of the KRAS signalling pathway.

To test the PITPNC1-regulated genes in a more clinically relevant setting, we performed GSEA using human LUAD data sets (*n* = 4) with information on the *KRAS* mutational status. A general enrichment of the dPITPNC1 GS was found in LUAD tumours harbouring *KRAS* mutations compared to those with native alleles (Fig. 3D). Likewise, we found a strong enrichment of the dPITPNC1 GS in human PDAC samples with regard to normal pancreas in two data sets (Fig. 3E). Additional analysis of genes whose expression was diminished in response to *PITPNC1* were recurrently present in the leading edges of the previously investigated data sets was done by qPCR. A dramatic reduction in mRNA expression was detected for all genes (Suppl. Figure 4A), validating the RNAseq data.

We next explored the clinical relevance of the dPITPNC1 GS. We observed that high dPITPNC1 GS levels were associated with the LUAD and PDAC patient subgroup with the worst prognosis (Fig. 3F-I). Analysis of the signature in the context of tumour stage revealed no differences in either tumour type (Suppl. Figure 4B and C). Likewise, no significant changes in two of the main patient subgroups of LUAD (mut *KRAS*/*P53*-mutated and mut *KRAS*/*LKB1*-mutated) and PDAC (classical and basal), which display differential outcome, response to therapy and gene expression profiles [41, 44, 45], were found (Suppl. Figure 4D and E). Collectively, these results indicate that PITPNC1 controls the expression of a gene signature with clinical implications for *KRAS*-mutated tumours.

### PITPNC1 loss induces a G1 phase arrest and MYC downregulation

**Fig. 4:**
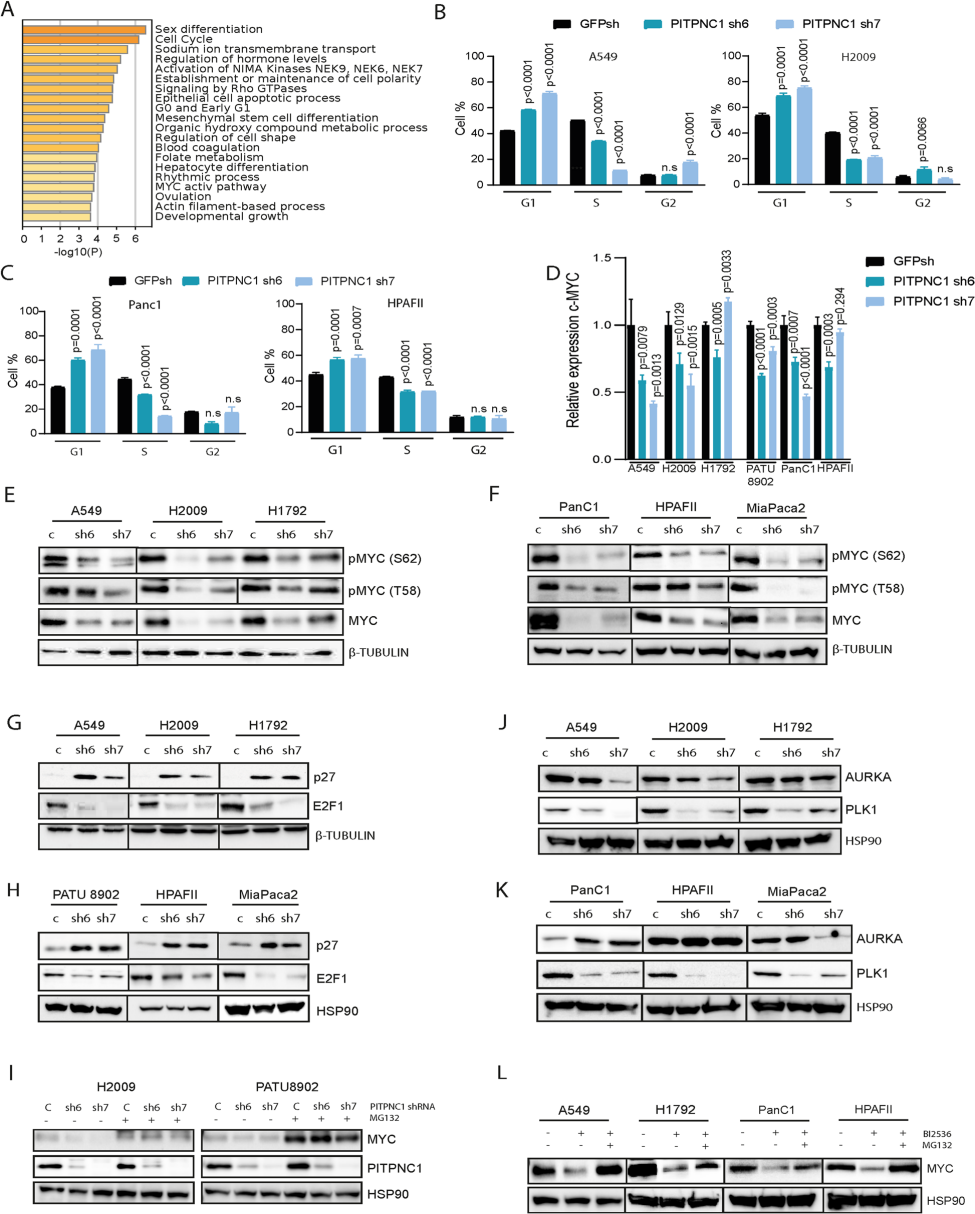
*PITPNC1* loss induces a G1 phase arrest linked to MYC downregulation. **A** Gene Ontology analysis of the downregulated *PITPNC1* gene set (dPITPNC1 GS). **B** and **C** Cell cycle analysis by EdU labelling in the human LUAD A549 and H2009 (B), and PDAC HPAFII and Panc1 (C) cell lines after *PITPNC1* knockdown with a specific shRNA (sh6 or sh7) compared to control (GFPsh). (Bonferroni´s multiple comparison test). **D ***MYC* mRNA expression in A549, H2009 and H1792 LUAD and PDAC PATU8902, Panc1 and HPAFII cell lines expressing a specific shRNA (sh6 or sh7) compared to control (GFPsh) (Dunnet´s multiple comparison test). **E** MYC protein expression in the A549, H2009 and H1792 LUAD cell lines after *PITPNC1* knockdown with a specific shRNA (sh6 or sh7) compared to control (*GFP*sh). Twenty μg of protein were loaded per sample. β-TUBULIN was used as loading marker. **F** MYC protein expression in Panc1, HPAFII and MiaPaca2 PDAC cell lines after *PITPNC1* knockdown with a specific shRNA (sh6 or sh7) compared to control (GFPsh). Twenty μg of protein were loaded per sample. β-TUBULIN was used as loading marker. **G** E2F1 and p27 protein expression in the A549, H2009 and H1792 LUAD cell lines after *PITPNC1* knockdown with a specific shRNA (sh6 or sh7) compared to control (GFPsh). Twenty μg of protein were loaded per sample. β-TUBULIN was used as loading marker. **H** E2F1 and p27 protein expression in the PATU8902, HPAFII and MiaPaca2 PDAC cell lines after *PITPNC1* knockdown with a specific shRNA (sh6 or sh7) compared to control (GFPsh). Twenty μg of protein were loaded per sample. HSP90 was used as loading marker. **I** MYC and PITPNC1 protein expression in H2009 and PATU8902 *MYC*-overexpressing cells after *PITPNC1* knockdown with a specific shRNA (sh6 or sh7) compared to control (GFPsh) and treated with DMSO or MG132 (10 μM, 6 h). Twenty μg of protein were loaded per sample. HSP90 was used as loading marker. **J** AURKA and PLK1 protein expression in A549, H2009 and H1792 LUAD cell lines after *PITPNC1* knockdown with a specific shRNA (sh6 or sh7) compared to control (GFPsh). Twenty μg of protein were loaded per sample. HSP90 was used as loading marker. **K** AURKA and PLK1 protein expression in Panc1, HPAFII and MiaPaca2 PDAC cell lines after *PITPNC1* knockdown with a specific shRNA (sh6 or sh7) compared to control (GFPsh). Twenty μg of protein were loaded per sample. HSP90 was used as loading marker. **L** MYC protein levels in A549 and H1792 LUAD and Panc1 and HPAFII PDAC cell lines treated with DMSO, PLK1i (BI2536, 50–100 nM, 48 h), or both PLK1i plus proteasome inhibitor (MG132, 10 μM 6 h). Twenty μg of protein were loaded per sample. HSP90 was used as loading marker

To expand our understanding of PITPNC1’s functional role in KRAS-driven oncogenesis, we performed Gene Ontology analysis to infer the biological pathways (BP) related to PITPNC1-regulated genes. First, the dPITPNC1 GS was used as input. The top BP included general cell cycle, sodium ion transmembrane transport, regulation of hormone levels or establishment/maintenance of cell polarity (Fig. 4A). These findings prompted us to inquiry about the impact of PITPNC1 loss on the cell cycle. We found a consistent G1 arrest and S phase decrease across all LUAD cell lines (Fig. 4B and Suppl. Figure 5A). These observations were extended to the PDAC setting (Fig. 4C), suggesting the regulation of common cellular mechanisms across mut *KRAS* tumours.

BP analysis also featured a MYC active pathway, which led us to test MYC expression in mut *KRAS* LUAD and PDAC cells with depleted *PITPNC1*. We found an overt MYC downregulation across all cell lines studied, which mainly occurred at the protein level (Fig. 4D-F), positioning MYC downstream of PITPNC1 and providing a direct link to the KRAS pathway. Such MYC downregulation was recapitulated upon *PITPNC1* inhibition in vivo (Suppl. Figure 5B). MYC cooperates with oncogenic RAS to regulate G1 to S phase transition of cell cycle [46], a phenotype observed in *PITPNC1*-depleted cells. Indeed, *MYC* inhibition using two specific shRNAs revealed a G1 arrest similar to that found in cells with *PITPNC1* loss (Suppl. Figure 5C). This mechanism involves repression of various cyclin kinase inhibitors, such as CDKN1B (p27) and CDKN1C (p57), and activation of E2F transcription factors [47], a link sustained in our experimental models (Suppl. Figure 5D). These observations led us to investigate the molecular consequences of *PITPNC1* loss on the cell cycle. Detailed analysis of transcriptomics data showed upregulation of p27 and p57, and downregulation of E2F1 (Suppl. Figure 5E). These results were validated using qPCR and Western blot analyses in independent samples (Fig. 4G and H, and Suppl. Figure 5F and G).

To investigate if PITPNC1 regulates cell cycle through MYC, exogenous *MYC* was overexpressed in *PITPNC1*-depleted cells. No rescue of the proliferative phenotype was found, most likely because MYC levels were still low even in the overexpressing cells (Suppl. Figure 5H and I). Notably, blocking the proteasome activity with the specific inhibitor MG132 rescued MYC expression, suggesting post-translational regulatory mechanisms (Fig. 4I). This prompted us to scan the *PITPNC1*-knockdown RNAseq data for potential kinases involved in MYC protein regulation, and found downregulation of *AURKA* and *PLK1* (Suppl. Figure 5J), two kinases previously reported to stabilize MYC protein via direct phosphorylation [48, 49]. Notably, only PLK1 was consistently decreased across the various LUAD and PDAC cell lines upon *PITPNC1* loss, with an expression pattern mimicking that of MYC protein (Fig. 4J and K). We next tested the possibility that PLK1 regulates MYC protein. Using the PLK1 inhibitor BI-2536, we found reduced MYC protein expression that is rescued by proteasome inhibition (Fig. 4L). Thus, PITPNC1 may be regulating MYC protein expression in part by PLK1. Taken together, these results suggest that PITPNC1 represents a functional link that connects oncogenic KRAS to MYC.

### PITPNC1 controls mTOR localization via MYC to prevent autophagy

**Fig. 5:**
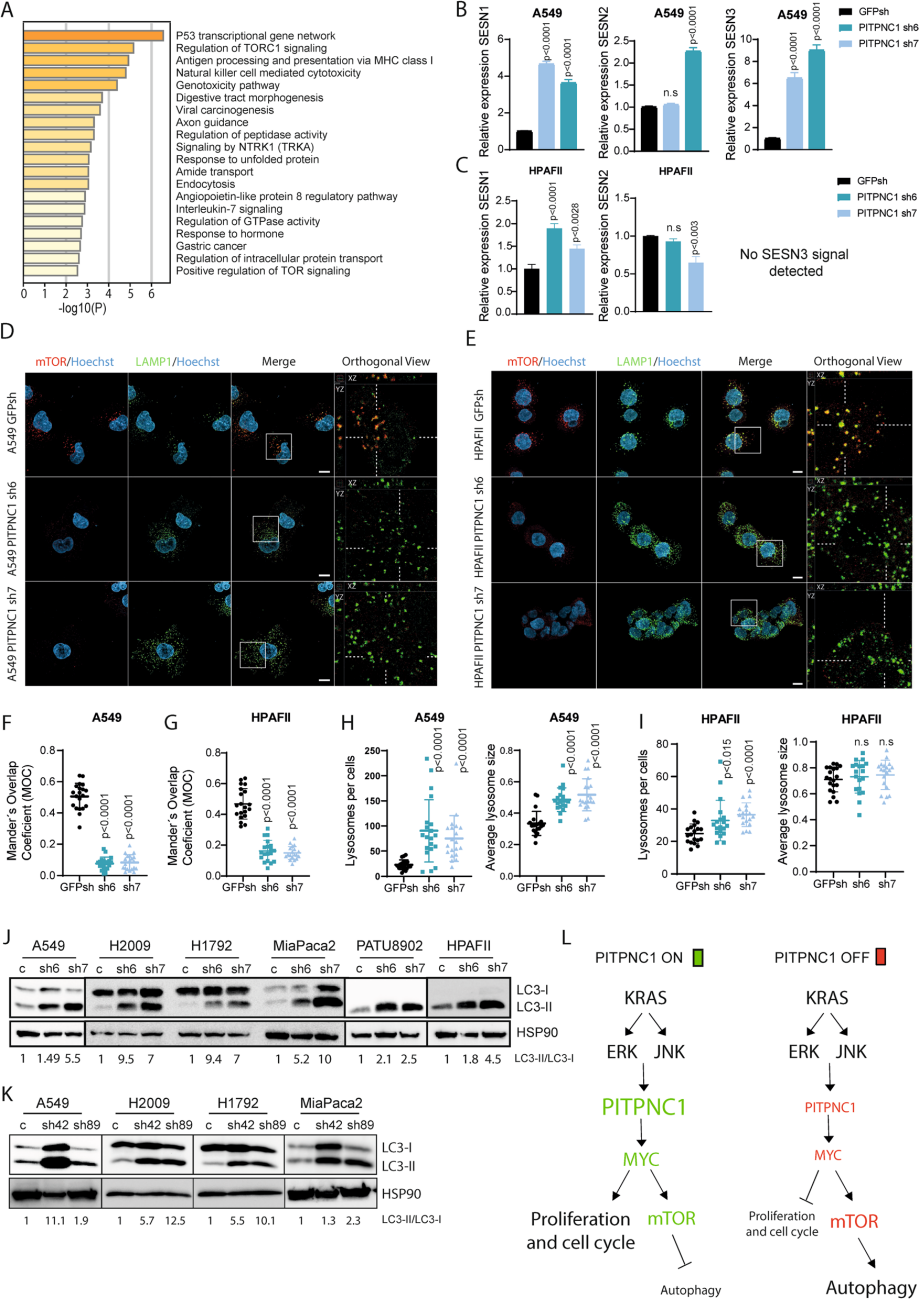
PITPNC1 controls mTOR localization to prevent autophagy. **A** Gene Ontology analysis of the upregulated *PITPNC1* gene set (uPITPNC1 GS). **B** and **C ***SESN1*, *SESN2* and *SESN3* expression levels in A549 (B) and HPAFII (C) cell lines were measured by qPCR. Cells were virally infected to express a control (*GFP*sh) or a *PITPCN1* shRNA (sh6 and sh7) (Dunnet’s multiple comparison test). GAPDH was used as housekeeping gene. **D** and **E** mTOR/LAMP1 colocalization analysis by immunofluorescence in A549 (D) and HPAFII (E) *PITPNC1*-depleted cells. **F** and **G** Quantification of mTOR/LAMP1 Mander’s overlap coefficient (MOC) in A549 (F) and (G) of D and E (Dunnett’s multiple comparison test). **H** Lysosomes per cell and average lysosomes size in A549 of D (Dunn’s multiple comparison test). **I** Lysosomes per cell and average lysosomes size in HPAFII of E (Dunn’s multiple comparison test). **J** Western blots of LC3-I and LC3-II protein levels in a LUAD (*n* = 3) and PDAC (*n* = 3) cell lines expressing a shRNA control (C) or two *PITPNC1* shRNAs (sh6 and sh7). Twenty μg of protein were loaded per sample and HSP90 was used as loading control. **K** Western blots of protein levels of LC3-I and LC3-II in a LUAD (*n* = 3) and PDAC (*n* = 1) cell lines expressing a shRNA control (C) or two *MYC* shRNAs (sh42 snd sh89). Twenty μg of protein were loaded per sample and HSP90 was used as loading control. **L** Proposed model for the role of PITPNC1 in KRAS-driven LUAD and PDAC

To complement the previous findings, we explored those genes upregulated upon *PITPNC1* abrogation (i.e., uPITPNC1 GS). The top 5 BPs of the GO analysis involved P53 transcriptional gene network, regulation of mTORC1 signalling, antigen processing and presentation of endogenous peptide antigen via MHC class I via ER pathway, natural killer cell-mediated toxicity, and genotoxicity pathway (Fig. 5A). We focused on mTOR as it is an effector of the PI3K pathway that can function within the KRAS signalling network. Enriched genes in the regulation of mTORC1 signalling BP feature included *CASTOR1*, *RRAGD*, *SESN1*, *SESN3*, and *GPR137C*. Upregulation of *SESN1*, *SESN2* and *SESN3* was validated at the mRNA level using qPCR and the results confirmed in additional *PITPNC1*-depleted cells (H2009 and HPAFII) (Fig. 5B and C, and Suppl. Figure 6A). These results suggested that activation of the mTOR pathway is altered upon *PITPNC1* inhibition.

SESTRINS (SESN1-3) inactivate GATOR2 to inhibit mTOR activity, constraining the localization of mTOR to the lysosome where it gets activated [50, 51]. Thus, we investigated mTOR localization in response to *PITPNC1* abrogation by immunofluorescence. A549, H2009 and HPAFII control cells showed mTOR activation, as inferred from the overlapping signal with the lysosome marker LAMP1. However, this colocalization was impaired when *PITPNC1* was inhibited (Fig. 5D-G, and Suppl. Figure 6B and C). This modification occurred without changes in mTOR protein abundance (Suppl. Figure 6D), suggesting that PITPNC1 controls mTOR lysosomal recruitment.

A close visualization of the lysosomes in *PITPNC1*-depleted cells revealed increased number and size compared to* PITPNC1*-proficient ones (Fig. 5H and I, and Suppl. Figure 6E). Expansion of the lysosomal compartment or lysosomal biogenesis has been related to enhanced autophagy [52]. mTOR functions as a counter-regulator of autophagy [53, 54]. Thus, we analysed the level of the autophagy marker LC3-II, which tightly correlates with the number of autophagosomes/autophagolysomes [55]. Increased LC3-II was observed in cell lines lacking PITPNC1 (Fig. 5J). In keeping with autophagy induction, downregulation of gene signature featuring autophagy and lysosome biogenesis [56] was also found in *PITPNC1*-inhibited cells (Suppl. Figure 6F).

Enhanced LC3-II expression could indicate either upregulation of autophagic flux (i.e., autophagosome formation) or blockade of autophagic degradation [57]. To confirm the underlying mechanism, we compared changes in LC3-II under the presence of the lysosomal protease inhibitor hydrochloroquine, which accumulates within lysosomes leading to lysosome neutralization and the inhibition of autophagic flux/autophagosome formation [55]. Hydroxichloroquine treatment elicited a further accumulation of LC3-II (Suppl. Figure 6G and H), indicating that PITPNC1 inhibition enhances autophagic flux. This mechanism occurred without activation changes in S6K and 4EBP1 (Suppl. Figure 6I).

MYC suppresses autophagy in B cell lymphomas by antagonizing the function of TFEB transcription factors [58], raising the possibility that PITPNC1 could control autophagy through MYC in LUAD and PDAC. To address this possibility, we first tested if MYC regulated autophagy in our experimental systems. MYC inhibition by specific shRNAs induced LC3II/I ratio in LUAD and PDAC cell lines (Fig. 5K). Autophagy induction was associated with reduced mTOR localization to lysosomes (Suppl. Figure 7A-D). This was associated with an increase in number and size of lysosomes (Suppl. Figure 7E and F). To define how MYC regulates autophagy, we tested if MYC could be transcriptionally controlling the negative regulators of mTOR localization, SESTRIN1-3, which are downregulated after *PITPNC1* inhibition. qPCR analysis of MYC-depleted cells showed that MYC inhibition significantly enhanced their expression (Suppl. Figure 7G and H), positioning SESTRINS downstream of MYC. Taken together, these observations suggest that PITPNC1 controls mTOR activity via MYC to prevent autophagy. A proposed model for the role of PITPNC1 in LUAD AND PDAC KRAS-driven tumours is depicted in Fig. 5L.

### JAK2 inhibitors reverse the expression of a *PITPNC1*-regulated transcriptome and synergize with Sotorasib

**Fig. 6:**
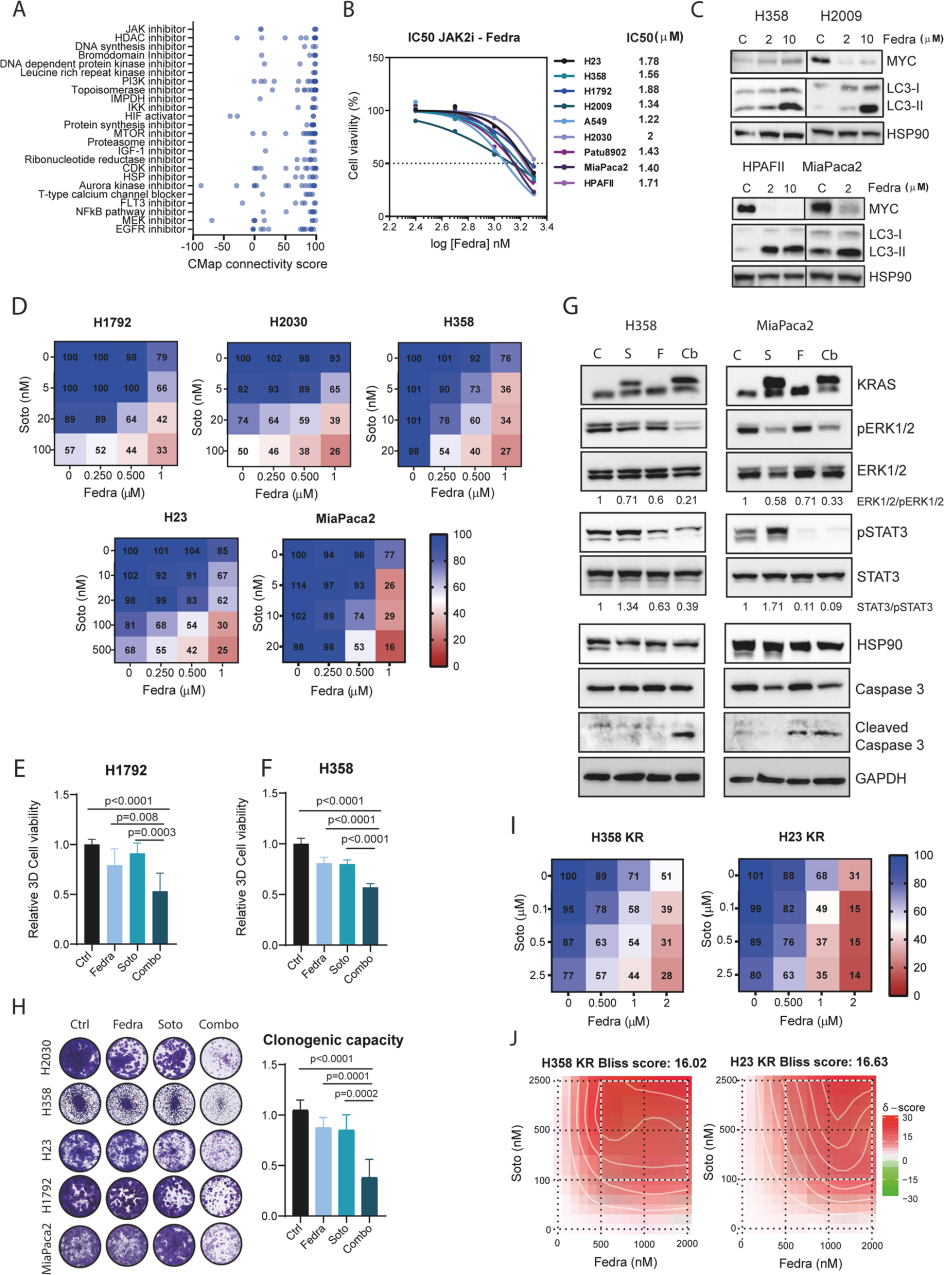
JAK2 inhibitors reverse the expression of the PITPNC1-regulated transcriptome and synergize with Sotorasib. **A** Connectivity Map (CMap) analysis of dPITPNC1 GS obtained in A549 cells. Perturbagen classes with mean connectivity scores > 90% are displayed. Each dot represents and individual drug included in the specific class.** B** Fedratinib (Fedra) IC50 index in a panel of LUAD and PDAC cell lines treated with the drug for 5 days. **C** Western blots of MYC and LC3-I/LC3-II in H358, H2009, HPAFII and MiaPaca2 treated with DMSO (C), and 2 or 10 μM of Fedra 48 h. Twenty μg of protein were loaded per sample and HSP90 was used as loading control. **D** Heatmaps of H1792, H2030, H358, H23 and MiaPaca2 cell viability percentage after treatment for 5 days with different concentrations of Sotorasib (Soto) and Fedra, individually or in combination. **E** and **F** Effects of Soto and Fedra combination on cell viability of mut *KRAS* LUAD cells (H1792 and H358) grown in 3D culture conditions, 5 days after drug treatment. Soto: 60 nM; Fedra: 1 μM. (Dunnett’s multiple comparison test). **G** Western blots of KRAS, pERK1/2, ERK1/2, pSTAT3, STAT3, HSP90, caspase 3, cleaved caspase 3 and GAPDH in H358 and MiaPaca2 cell lines treated with vehicle (Ctrl), 20 nM Soto, 1 μM Fedra, or both (Combo) for 48 h. Twenty μg of protein were loaded per sample. HSP90 and GAPDH were used as loading controls. **H** Representative image and quantification of clonogenic capacity of H2030 (Soto: 5 nM; Fedra: 0.5 µM), H358 (Soto: 5 nM; Fedra: 0.25 µM), H23 (Soto: 5 nM; Fedra: 0.5 µM) H1792 (Soto: 20 nM; Fedra: 0.5 µM) and MiaPaca2 (Soto: 20 nM; Fedra: 0.5 µM) cells treated with the indicated drugs and concentrations for 10 days, (Dunnett’s multiple comparison test). **I** Heatmaps showing cell viability percentage of Soto-resistant (SR) H23 and H358 cell lines treated with different concentrations of Soto and Fedra, individually or in combination. **J** Synergistic score (Bliss score) heatmaps of Soto-resistant (SR) H23 and H358 cells treated for 5 days as indicated

Given the lack of pharmacological tools to inhibit PITPNC1 and aiming to increase the translational value of our findings, we followed a drug repurposing strategy that predicts compounds capable of reversing the expression profile of the PITPNC1-regulated transcriptome. The top 200 up and down differentially expressed genes obtained after *PITPNC1* knockdown (logFC ± 1, B > 0) were used as input and a repurposing score > 90 was used as cut-off. The top 5 drug families predicted to reverse the PITPNC1 transcriptome were JAK, HDAC, DNA synthesis, bromodomain and DNA dependent protein kinase inhibitors (i). Additional drug families scoring in this analysis were PI3Ki, mTORi or MEKi, known downstream effectors of KRAS oncogene (Fig. 6A). The same drug repurposing approach applied to a KRAS-dependent transcriptome uncovered common drug families (Suppl. Figure 8A), consistent with the overlap of PITPNC1- and KRAS-regulated genes. JAK inhibitors, particularly those against JAK2, scored highest in both repurposing studies and were selected for downstream analyses.

To investigate if JAK2 inhibitors would phenocopy to some extent the effect caused by PITPNC1 abrogation, LUAD and PDAC cell lines were treated with Fedratinib, a highly specific JAK2i. A gradual decrease of cell proliferation was observed with increasing concentrations of Fedratinib in all cell lines (Fig. 6B). IC50 values ranged from 1.2 to 2 µM. Of note, Fedratinib treatment induced MYC depletion, LC3 upregulation or both, partially recapitulating the PITPNC1 inhibition phenotype (Fig. 6C). Thus, JAK2 inhibitors could function as a surrogate tool of PITPNC1 depletion.

KRASG12C inhibitors have emerged as promising targeted agents for mut *KRAS*-driven tumours [59, 60], albeit the clinical data suggest that combinatorial strategies may be required for more durable antitumour responses [61–63]. A rational concept for combination therapies builds on maximal driver pathway inhibition [64]. Thus, we tested the combination of the KRASG12Ci Sotorasib with Fedratinib, both approved by the Food and Drug Administration (FDA) [65, 66]. To do this, human *KRASG12C* LUAD and PDAC cell lines (*n* = 5) were treated alone or in combination with various concentrations < IC25 of each drug. A larger antiproliferative phenotype was elicited by the drug combination compared to individual drugs (Fig. 6D and Suppl. Figure 8B). SynergyFinder revealed that the dual treatment was synergistic (Suppl. Figure 8C). The drug combination was also studied in 3D organoid cultures, given the enhanced KRAS oncogene dependence observed compared to 2D cultures [67, 68]. Likewise, the dual treatment effected cell proliferation more largely than single drugs (Fig. 6E and F, Suppl. Figure 8D). Molecular characterization of downstream targets revealed activation of STAT3 in response to KRASG12C inhibition that was suppressed in the drug combination. Furthermore, specific apoptosis induction was also observed in the combined treatment, suggesting a cytotoxic effect (Fig. 6G).

Next, we assessed the effect of the Fedratinib-Sotorasib combination in the context of adaptive resistance. First, using a 10 day-treatment colony forming assay, where early adaptive, non-genetic resistant mechanisms are likely to arise in response to individual drugs, we found that both drugs restrict cell proliferation to a greater extent than drugs alone (Fig. 6H). Second, the dual combination was tested in cell lines that had been made resistant to Sotorasib through gradual treatment with increasing drug concentrations for over 1 month (H358SR and H23SR) (Macaya and Roman et al., under review). The combined treatment yielded an antiproliferative response that was higher than each drug in the two resistant cells and also synergistic (Fig. 6I and J, and Suppl. Figure 8E). Thus, a JAK2i potentiates KRASG12Ci’s effect in both treatment-naïve and KRASG12Ci-resistant cells.

### Anti-tumour activity of combined JAK2 and KRASG12C inhibitors in vivo

**Fig. 7:**
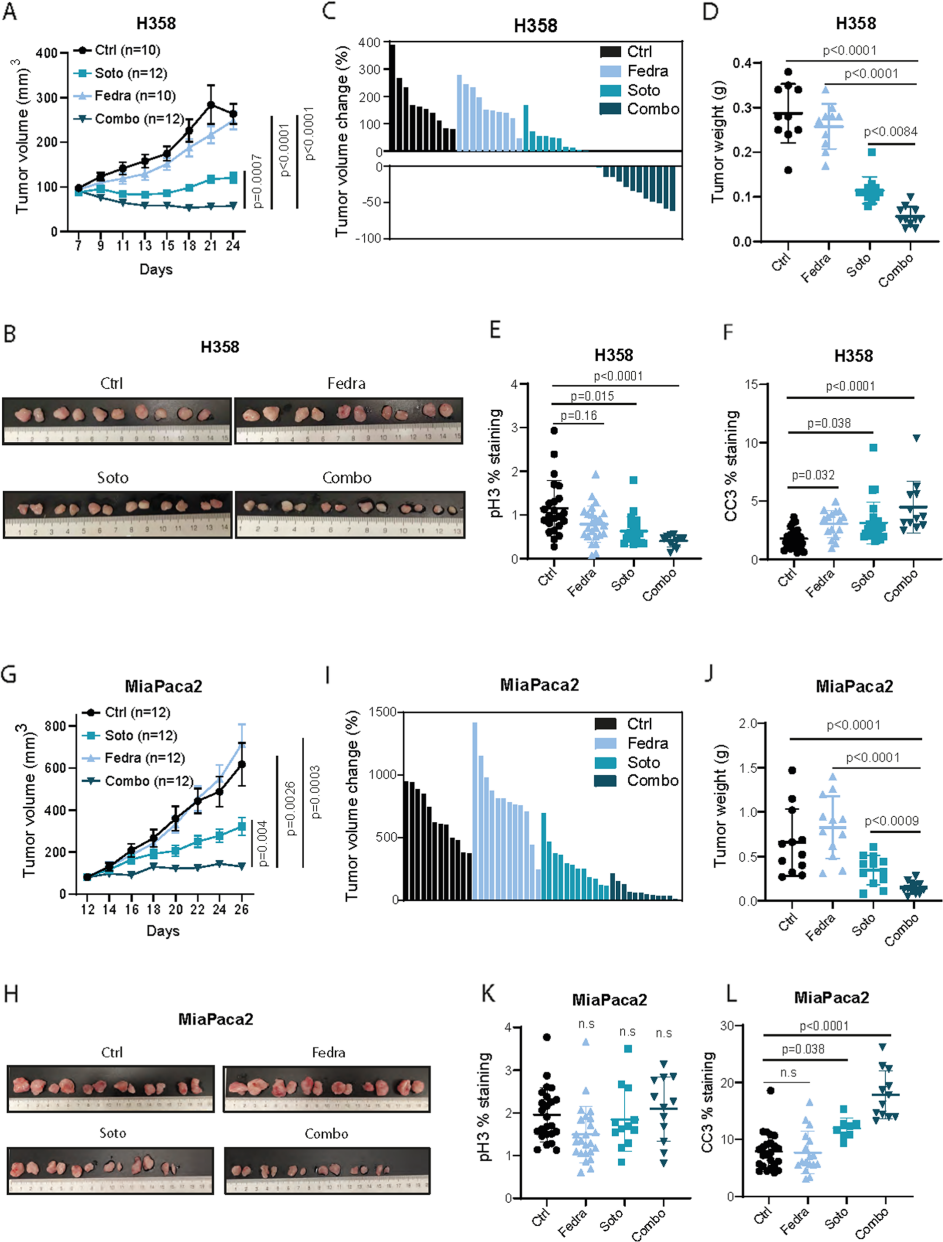
Antitumour activity of combined JAK2 and KRASG12C inhibitors in vivo. **A** Tumour volume (mm^3^) of cell-derived tumours from H358 cells treated with indicated drugs (Sotorasib -Soto-: 10 mg/kg once daily; Fedratinib -Fedra-: 60 mg/kg twice daily). *n* = 10–12 tumours per group (Tukey’s multiple comparison test). **B** Representative images of tumours in A. **C** Waterfall plots of cell-derived tumours from H358 cells at the last day of experiment after being treated with the indicated drugs. **D** Tumour weight (g) of H358 cell-derived tumours of the tumours of A at end point (Dunnett’s multiple comparison test). **E** and **F** pH3 (E) and CC3 (F) quantification of H358 derived xenografts at end point (Dunnett’s multiple comparison test). **G** Tumour volume (mm^3^) of tumours derived from MiaPaca2 cells treated with indicated drugs (Soto: 10 mg/kg once daily; Fedra: 60 mg/kg twice daily). *n* = 12 tumours per group, (Tukey’s multiple comparison test). **H** Representative images of tumours in G. **I** Waterfall plots of cell-derived tumours from MiaPaca2 cells at the last day of experiment after being treated with the indicated drugs. **J** Tumour weight (g) of MiaPaca2 cell-derived tumours of the tumours from I (Dunnett’s multiple comparison test). **K** and **L** pH3 (E) and CC3 (F) quantification of MiaPaca2-derived xenografts at end point (Dunnett’s multiple comparison test)

To investigate the impact of the dual combination in a more physiologically representative system, we generated cell-derived xenograft (CDX) models from LUAD (H358) and PDAC (MiaPaca2) cell lines in immunodeficient mice (*n* = 10–12 group). Fedratinib was administered at 60 mg/kg (twice daily) and Sotorasib at suboptimal concentrations 10 mg/kg (daily) as described earlier [59]. Treatment started when the average volume of H358- and MiaPaca2-derived tumours reached 80 or 90 mm^3^ respectively. In the H358 CDX model, tumours treated with Fedratinib displayed growth kinetics similar to the vehicle-treated group, while those treated with Sotorasib were much lower, with a slight volume increase over time. Notably, concomitant drug administration led to generalized tumour regressions (Fig. 7A-D). Analysis of cell proliferation (pH3) and apoptosis (CC3) 7 days post-treatment revealed a notable decrease and increase respectively upon combined treatment (Fig. 7E and F, and Suppl. Figure 9A). In the MiaPaca2 CDX model, Fedratinib had no impact on tumour growth as compared to control mice whereas Sotorasib delayed tumour growth. The drug combination had a more profound impact on tumour growth, with tumour volume barely changing from the start point. The overt effect of the drug combination on the tumour volume translated into significantly smaller tumours (Fig. 7G-J). Histological analysis 7 days post-treatment showed increased apoptosis but no changes on tumour proliferation after the dual treatment (Fig. 7K and L, and Suppl. Figure 9B). More importantly, the effect of the drug combination had no consequences on the mice weight (Suppl. Figure 9C and D).

Collectively, these results nominate the combination of JAK2 and KRASG12C inhibitors as a potential strategy to treat KRAS-driven LUAD and PDAC harboring *G12C* mutation.

## Discussion

Through a series of clinical, cellular, molecular, and in vivo analyses, our study provides the first evidence that the phospholipid transporter PITPNC1 stands as a functional KRAS effector in LUAD and PDAC, further confirming the critical role of the phospholipid transport machinery in cancers driven by the KRAS oncogene.

PITPNC1 functions as a phospholipid transporter that was originally linked to the metastatic phenotype in breast cancer [17]. However, while subsequent studies using PITPNC1 staining of primary gastric and rectal cancer revealed an association with clinical stage and poor prognosis, and radio-resistance respectively [19], the involvement of PITPNC1 in the regulation of additional cellular and molecular mechanisms in cancer was still unknown. We provide data indicating that PITPNC1 consistently controls the cell cycle of LUAD and PDAC driven by oncogenic KRAS, in part due to the regulation of positive (E2F1) and negative (P27, P57) cell cycle modulators by the transcription factor MYC. MYC protein expression consistently decreases in the PITPNC1 loss phenotype, a mechanism that involves PLK1 downregulation, a kinase that stabilizes MYC protein via direct phosphorylation [49]. Indeed, PLK1 or proteasome pharmacological inhibition rescues MYC expression. In addition to MYC protein stabilization via ERK1/2 phosphorylation [69–71] under normal conditions, or through ERK5 when cancer cells are treated with MEK1/2 or ERK1/2 inhibitors [72, 73], our data provides a new mechanism for MYC protein regulation by KRAS oncogene.

We find that PITPNC1 loss also exacerbates the antiproliferative effect by inducing autophagy across mut *KRAS* LUAD and PDAC. This function depends on the unexpected regulation of mTOR activity via control of its cellular localization, as *PITPNC1*-deficient mut *KRAS* cells display a dramatic mTOR delocalization from lysosomes. This mechanism seems to be dependent on MYC activity, as MYC depletion phenocopies the autophagy induction elicited by PITPNC1 inhibition. A plausible explanation to mTOR delocalization may be the consistent upregulation of *SESTRIN1-3* observed in the RNAseq and qPCR data. SESTRINS have been reported to control mTORC1 localization and subsequent activity via negatively regulation of the amino acid sensing pathway upstream of mTORC1 through GATOR2 binding [50], a mechanism that is partially phenocopied by *PITPNC1* loss. Alternatively, PITPNC1’s canonical role regulating PA availability as a lipid transporter could also explain mTOR activation. Indeed, phospholipase D-dependent accumulation of cellular PA after mitogenic stimulation is required for phosphorylation of mTOR downstream effectors S6K and 4EBP1 [74]. While we cannot completely rule out that the canonical function of PITPNC1 may also influence mTOR activity, we have not detected consistent activation of S6K and 4EBP1 in *PITPNC1*-depleted conditions that would support such activation mechanism. In any case, our data highlight a novel mechanism whereby KRAS can regulate mTOR activation via MYC, what adds to the previous reports showing that mTOR activation by the KRAS pathway occurred via phosphorylation by the ERK-RSK axis [75–77].

The cellular and molecular data highlighting the contribution of PITPNC1 to KRAS oncogenesis are supported by complementary analyses using LUAD and PDAC clinical data. First, PITPNC1 is uncovered as a marker of poor prognosis in both tumour types. In tune with these data, a PITPNC1-downregulated gene signature showed an inverse correlation with overall survival. The consistent results across tumours suggest that a PITPNC1-regulated network represents a relevantly common signalling node in KRAS oncogenesis with clinical implications that could be exploited for therapeutic purposes.

Studies focused on PITPNC1 have been limited by the lack of pharmacological inhibitors, which precluded exploring its role as a molecular target in cancer or other diseases. Our study supports the use of gene expression-based drug repurposing to infer drugs that could recapitulate the gene expression network controlled by a particular gene. This approach unveiled the family of JAK inhibitors in general and JAK2i in particular among the top predicted drugs in addition to PI3K, mTOR, or MEK1/2 inhibitors, known downstream effectors of KRAS oncogene. The fact that mTOR inhibitors are predicted to control a PITPNC1 signature further supports the link between mTOR and the phospholipid transporter. Of note, drug predictions largely overlapped with those obtained using a KRAS-regulated gene signature, suggesting that JAK inhibitors may indeed function in mut *KRAS* cancer. In keeping with this possibility, LUAD and PDAC lines are sensitive to the JAK2i Fedratinib at the low micromolar range. Although JAK2 inhibitors have only been approved for the treatment of myeloproliferative disorders [78], several preclinical studies have shown the implication of the JAK/STAT pathway in solid tumours [79–81]. Corcoran et al. demonstrated how activation of the JAK/STAT pathway is critical for the maintenance and development of PDAC [82]. Furthermore, the relevance of this pathway was also reported in NSCLC [83], pulmonary fibrosis [84], or colorectal cancer [85, 86]. Collectively, these findings nominate JAK2 inhibitors as potential drugs for the treatment of KRAS-driven cancers.

Given that single drugs elicit limited antitumour responses in *KRAS*-mutated cancers, in part due to resistance mechanisms, this study proposes the combination of JAK2i with the recently FDA-approved KRASG12Ci Sotorasib based on a notable synergistic effect in vitro and in vivo. Testing this drug combination is mechanistically supported by the fact that the JAK/STAT pathway can act as a mechanism of compensation to MAPK pathway inhibition treatment [87]. Furthermore, concomitant JAK2 and MEK1/2 inhibition reprograms the cancer-associated fibroblast (CAF) and immune microenvironment to overcome resistance to anti-PD-1 therapy in PDAC [88]. These observations warrant the exploration of the JAK2i-KRASG12Ci drug combination alone or in the context of immune checkpoint inhibitors. The fact that both drugs are already approved for clinical applications and known to be well-tolerated may facilitate their progression to clinical trials. Lastly, while our observations are restricted to combinations using a KRASG12Ci, it is tempting to speculate that upcoming KRAS inhibitors targeting alternative mutations (e.g. G12D or G12V) may also synergize with JAK2 inhibitors, opening a new avenue for the treatment of a larger fraction of LUAD and PDAC patients.

## Conclusions

In conclusion, our work uncovers the phospholipid transporter PITPNC1 as a KRAS effector that controls central transcriptional and signalling nodes (i.e. MYC and mTOR) and unveils novel therapeutic strategies for KRAS-driven tumours in the context of a PITPNC1-regulated transcriptional network.

## Supplementary Information

**Additional file 1: Suppl. Figure 1.** A. *PITPNC1* expression levels (log2) in TCGA LUAD patient’s database. Mut EGFR: mutant *EGFR*, wt: wild type *EGFR*, N: Normal tissue. Mut vs wt (p=0.014), Mut vs N (p=0.021). B. *PITPNC1* expression levels (log2) in TCGA LUAD patient’s database. Mut BRAF: mutant *BRAF*, wt: wild type *BRAF*, N: Normal tissue. Mut vs wt (p=0.275), Mut vs N (p=0.336). C. *PITPNC1* expression levels (log2) in TCGA LUAD patient’s database with different KRAS point mutations. N: Normal tissue D. Western blot of PITPNC1 and KRAS expression in H1792 cells, expressing a control (GFPsh) or a tet-inducible *KRAS* shRNA (KRASsh) (activated by 1 μg/ml doxycycline). Twenty μg of protein were loaded per sample. β-TUBULIN was used as loading markes E. *PITPNC1* mRNA expression in H2009 and H1792 cells expressing a control (GFPsh) or a tet-inducible *KRAS* shRNA (KRASsh) (activated by 1 μg/ml doxycycline) (Mann-Whitney or unpaired t-test). F. Western blot of pERK1/2, ERK1/2, pAKT, AKT, p-cJUN, cJUN, pERK5 and ERK5 in A549, H2009 and HPAFII treated with pharmacologic inhibitors: trametinib (MEKi, 0.5 μmol/L), BIX02189 (MEK5i, 10 μmol/L) or GSK2126458 (PI3Ki, 0.1 μmol/L) for 24 h, and SP600125 (JNKi, 10 μmol/L) for 2 h. Twenty μg of protein were loaded per sample. G. *PITPNA, PITPNB, PITPNM1, PITPNM2, PITPNM3* mRNA expression in H2126 cells overexpressing a mutant (KRASG12D) or a wild type form (KRAS4B) of *KRAS* compared to the control (LacZ) (Dunnett’s multiple comparison test). H and I. *PITPNA, PITPNB, PITPNM1, PITPNM2, PITPNM3* mRNA expression in H2009 (G) and H1792 (H) cells expressing a control (GFPsh) or an inducible *KRAS* shRNA (KRASsh) (activated by 1 μg/ml doxycycline) (Mann-Whitney or unpaired t-test). J. Western blot of PITPNC1 and KRAS expression in *Kras*^lox/lox^ MEFs transduced with different human HA-tagged KRAS mutants (G12C, G12D, G12V, G12R, G12S, G13D and Q61H). K. *PITPNC1* expression levels (log2) in TCGA LUAD patient’s database. Mut KRAS: mutant *KRAS*. Mut KRAS Amp: mutant *KRAS* amplification, N: Normal tissue. Mut KRAS vs Mut KRAS Amp (n.s). **Suppl. Figure 2.** A. *PITPNC1* expression levels (log2) in TCGA LUAD patient’s database in presence of a panel of co-occurrence mutations. B. Western blot of LKB1 and PITPNC1 expression in H2009 cells expressing a control (Control) or *LKB1* sgRNAs (*LKB1* sgRNA1 or *LKB1* sgRNA2). Twenty μg of protein were loaded per sample. β-TUBULIN was used as loading marker. C. Western blot of PITPNC1, p53, Keap1 or Lkb1 expression in KLA and LKR10 cells, expressing a control (Control) or *p53*, *Lkb1* or *Keap1* sgRNAs (p53 sgRNA, Lkb1 sgRNA or Keap sgRNA). Twenty μg of protein were loaded per sample. HSP90 and β-TUBULIN were used as loading markers. **Suppl. Figure 3.** A. Apoptosis analysis by Annexin V/7AAD labelling in the human LUAD A549, H2009, H1792 and H358 cell lines after *PITPNC1* knockdown with a specific shRNA (sh6 or sh7) compared to the control (GFPsh). (Tukey’s multiple comparison test). B. Representative flow cytometry images depicting gating strategy in A. C. Representative images of A549- and PATU8902-derived xenografts (GFPsh, PITPNC1 sh6 and PITPNC1 sh7) stained for phospho-histone 3 (pH3) or cleaved caspase 3 (CC3). D. Western blot of *PITPNC1* in A549 and H358 cells transfected with a control (pBabe) or *PITPNC1* cDNA. Twenty μg of protein were loaded per sample. β-TUBULIN was used as loading marker E. Representative images of clonogenic ability of A549 and H358 *PITPNC1*-overexpressing cells compared with the control. F. Tumour volume (mm^3^) of A549 derived xenografts, (n=8), (Bonferroni’s multiple comparison test). G. Representative images of tumours from F. H. Tumor weight (g) of tumours in F (n=8) (Mann-Whitney or Unpaired T-test). I. Tumour volume (mm^3^) of H358 derived xenografts, (n=8), (Bonferroni’s multiple comparison test). J. Representative images of tumours from I. K. Tumour weight (g) of tumours in I (n=8) (Mann-Whitney or Unpaired T-test). L. Migration assay experiment in *LacZ* and *PITPNC1*-overexpressing A549 and H2009 cell lines. M. Representative bioluminescence images of a metastasis assay via intracardiac injection of *LacZ* and *PITPNC1*-overexpressing A549 cells. N. *Ex vivo* analysis of bioluminescence in lung, liver and kidney. **Suppl. Figure 4.** A. *PHLDA2, GJB2, GPX2, BIRC5, PITPNC1, RASSF6* and *ARK1B10* mRNA expression in A549 cells expressing a control (GFPsh) or a *PITPNC1* shRNA (sh6 or sh7) (Dunnett´s multiple comparison test). B. Box plot comparing early (I-II) and advanced (III-IV) mut *KRAS* LUAD according to the expression of the dPITPNC1 gene signature (GS). C. Box plot comparing localized and advanced (locally advanced and metastatic) PDAC based on the expression of the dPITPNC1 gene signature (GS). D. Box plot comparing *P53*-mutated and LKB1-mutated LUAD patients with mut *KRAS* according to the expression of the dPITPNC1 gene signature (GS). E. Box plot comparing classical and basal PDAC patients based on the expression of the dPITPNC1 gene signature (GS). **Suppl. Figure 5.** A. Cell cycle analysis by EdU labelling in the human LUAD H1792 and H358 cell lines after *PITPNC1* knockdown with a specific shRNA (sh6 or sh7) compared to the control (GFPsh) (Bonferroni´s multiple comparison test). B. Western blot of MYC expression in A549- and PATU8902-xenografts tumours. Twenty μg of protein were loaded per sample. HSP90 and GAPDH were used as loading markers. C. Cell cycle analysis by EdU labelling in A549 and Miapaca2 cell lines after *PITPNC1* knockdown with a specific shRNA (sh6 or sh7) compared to the control (GFPsh) (Dunnet´s multiple comparison test). D. Western blot of MYC, E2F1 and P27 in A549, H2009, H1792 and Miapaca2 cells, expressing a control (GFPsh) or a shRNA against *PITPNC1* (sh6 or sh7). Twenty μg of protein were loaded per sample. β-TUBULIN was used as loading control. E. mRNA analysis of RNAseq data of *E2F1*, *P27* and *P57* in A549 cells expressing a control (GFPsh) or a shRNA against *PITPNC1* (sh6 or sh7). F and G. QPCR analysis of *P27* (F) and P57 (G) mRNA expression in A549, H2009, H1792 and PATU8902 cells expressing a control (GFPsh) or a *PITPNC1* shRNA (sh6 or sh7) (Dunnett´s multiple comparison test). H. Cell proliferation assay in A549 and H2009 cells expressing exogenous *LacZ* or *MYC*- and submitted to inhibition of PITPNC1 by specific shRNAs. I. Western blot of MYC, PITPNC1 in A549 and H2009 cells expressing exogenous *LacZ* or MYC- and submitted to inhibition of PITPNC1 by specific shRNAS. Twenty μg of protein were loaded per sample. HSP90 was used as loading control. J. RNAseq data of AURKA and PLK1 in A549 expressing a control (GFP) or two *PITPNC1* shRNAs. **Suppl. Figure 6.** A. *SESN1*, *SESN2* and *SESN3* expression levels in H2009 cell line were measured by qPCR. Cells were virally infected to express a control (*GFP*sh) or a *PITPCN1* shRNA (sh6 and sh7) (Dunnet´s multiple comparison test). GAPDH was used as housekeeping gene. B. mTOR/LAMP1 colocalization analysis by immunofluorescence in H2009 *PITPNC1*-depleted cells. C. Quantification of mTOR/LAMP1 Mander´s overlap coefficient (MOC) in H2009 of B (Dunnett’s multiple comparison test). D. Western blot of mTOR in A549, H2009 and H1792 cell lines expressing a control (GFP) or two *PITPNC1* shRNAs. Twenty μg of protein were loaded per sample and HSP90 was used as loading control. E. Lysosomes per cell and average lysosomes size in H2009 of B (Dunn’s multiple comparison test). Lysosomes per cell and average lysosomes size in H2009 of B (Dunn’s multiple comparison test). F. Heatmap of autophagy and lysosome biogenesis genes upregulated upon PITPNC1 inhibition in A549 cells (data from RNAseq analysis). G and H. Western blot of protein level of LC3-I and LC3-II in A549, H2009 (G) and HPAFII (H) virally infected to express a shRNA control (C) or two *PITPNC1* shRNAs (sh6 and sh7) and treated with or without hydroxychloroquine (CQ) (60 μM) for 6 h. Twenty μg of protein were loaded per sample and HSP90 was used as loading control. I. Western blot of mTOR signalling pathway (mTOR, 4EBP1 and S6K) in A549, H2009 and H1792 cell lines in which *PITPNC1* was inhibited with specific shRNAs (sh6 and sh7). Twenty μg of protein were loaded per sample and HSP90 was used as loading control. **Suppl. Figure 7.** A. mTOR/LAMP1 colocalization analysis by immunofluorescence in A549 *MYC*-depleted cells. B. Quantification of mTOR/LAMP1 Mander´s overlap coefficient (MOC) in A549 of A (Dunnett’s multiple comparison test). C. mTOR/LAMP1 colocalization analysis by immunofluorescence in A549 *MYC*-depleted cells. D. Quantification of mTOR/LAMP1 Mander´s overlap coefficient (MOC) in MiaPaca2 *MYC*-depleted cells (unpaired t-test). E. Lysosomes per cell in A549 and MiaPaca2 of A and C respectively (Dunn’s multiple comparison test and unpaired t-test respectively). F. Average lysosomes size in A549 and MiaPaca2 of A and C respectively (Dunn’s multiple comparison test and unpaired t-test respectively). G. *SESN1*, *SESN2* and *SESN3* expression levels in A549 cell line were measured by qPCR. Cells were virally infected to express a control (*GFP*sh) or a *MYC* shRNA (sh42 and sh89) (Dunnet´s multiple comparison test). GAPDH was used as housekeeping gene. H. *SESN1*, *SESN2* and *SESN3* expression levels in Miapaca2 cell line were measured by qPCR. Cells were virally infected to express a control (*GFP*sh) or a *MYC* shRNA (sh42 and sh89) (Dunnet´s multiple comparison test). GAPDH was used as housekeeping gene. **Suppl. Figure 8.** A. Connectivity Map (CMap) analysis for dKRAS GS H358 transcriptomics. Perturbagen classes with mean connectivity scores >90% are displayed. Each dot represents an individual drug included in the specific class. B. Representative image of crystal violet stained plates for drug combination experiment in G12C cell lines. C. Synergistic score (Bliss score) heatmaps of H1792, H2030, H358, H23 and MiaPaca2 treated for 5 days as indicated D. Representative image of 3D proliferation assay in H358 and H1792 cell lines treated with DMSO (Ctrl) Soto (60 nM) Fedra (1 μM) or both (Combo) for 5 days. E. Representative image of crystal violet stained plates for drug combination experiment in Soto-resistant (KR) G12C cell lines H358 and H23. **Suppl. Figure 9.** A and B. Representative images of H358- and Miapaca2-derived xenografts stained for phospho-histone 3 (pH3) or cleaved caspase 3 (CC3). C and D. Mouse weight change upon different treatments. S= start of the experiment; E= end of the experiment (Mann-Whitney or unpaired t-test).

## Data Availability

The datasets generated and/or analysed during the current study are available through the referenced publications or at GEO website as described in the Methods section.

## Abbreviations

4EBP1: Eukaryotic translation initiation factor 4E binding protein 1
ARID1A: AT-rich interaction domain 1A
AURKA: Aurora kinase A
BP: Biological pathways
BRAF: V-raf murine marcoma viral oncogene homolog B1
CAF: Cancer-associated fibroblast
CASTOR1: Cytosolic arginine sensor for MTORC1 subunit 1
CCND1: Cyclin D1
CDX: Cell-derived xenograft
dPITPNC1 GS: Downregulated PITPNC1 gene signature
DUSP4: Dual specificity phosphatase 4
DUSP6: Dual specificity phosphatase 6
E2F1: E2F transcription factor 1
EGFR: Epidermal growth ractor receptor
GATOR2: GATOR complex protein 2
GEM: Genetically engineered mouse
ICGC: International Cancer Genome Consortium
JAK2: Janus kinase 2
JNK: C-Jun N-terminal kinase
KEAP1: Kelch-like ECH associated protein 1
KRAS: Kirsten rat sarcoma viral oncogene homologue
LAMP1: Lysosomal associated membrane protein 1 or CD107a
LC3-II: Microtubule associated protein 1 light chain 3 alpha/beta
LKB1: Liver kinase B1
LUAD: Lung adenocarcinoma
MAPK: Mitogen-activated protein kinase
MEK: Mitogen-activated protein kinase
mTOR: Mammalian target of rapamycin kinase
Mut: Mutant
MYC: CMYC proto-oncogene, bHLH transcription factor
ORP5: Oxysterol Binding Protein Like 5
ORP8: Oxysterol Binding Protein Like 8
P27: Cyclin dependent kinase inhibitor 1B or CDKN1B
P53: Tumour protein 53 (TP53)
P57: Cyclin dependent kinase inhibitor 1C or CDKN1C
PA: Phosphatidic acid
PC: Phosphatidylcholine
PDAC: Pancreatic ductal adenocarcinoma
PHLDA1: Pleckstrin homology-like domain family A member
PI: Phosphatidylinositol
PI3K: Phosphatidyl inositol 3-kinase
PIP3: Phosphatidylinositol (3,4,5)-trisphosphate
PITP: Phosphatidylinositol transfer protein
PITPNA: Phosphatidylinositol transfer protein α
PITPNB: Phosphatidylinositol transfer protein β
PITPNC1: Phosphatidylinositol transfer protein cytoplasmatic 1
PITPNM1: Phosphatidylinositol Transfer Protein Membrane Associated 1
PITPNM2: Phosphatidylinositol Transfer Protein Membrane Associated 2
PITPNM3: Phosphatidylinositol Transfer Protein Membrane Associated 3
PLK1: Polo-like kinase 1
PS: Phosphatidylserine
S6K: Ribosomal protein S6 kinase
SESN1: Sestrin 1
SESN2: Sestrin 2
SESN3: Sestrin 3
SgRNA: Single-guide RNA
SPRY4: Sprouty homolog
STAT: Signal transducer and activator of transcription
TCGA: The Cancer Genome Atlas
TSGs: Tumour suppressor genes
uPITPNC1 GS: Upregulated PITPNC1 gene signature
Wt: Wild type
