# Correction: The phospholipid transporter PITPNC1 links KRAS to MYC to prevent autophagy in lung and pancreatic cancer

**DOI:** 10.1186/s12943-023-01795-x

**Published:** 2023-06-16

**Authors:** Rodrigo Entrialgo‑Cadierno, Cristina Cueto‑Ureña, Connor Welch, Iker Feliu, Irati Macaya, Laura Vera, Xabier Morales, Sandra Vietti Michelina, Pietro Scaparone, Ines Lopez, Elodie Darbo, Oihane Erice, Adrian Vallejo, Haritz Moreno, Ainhoa Goñi‑Salaverri, David Lara‑Astiaso, Nils Halberg, Ivan Cortes‑Dominguez, Elizabeth Guruceaga, Chiara Ambrogio, Fernando Lecanda, Silve Vicent

**Affiliations:** 1 Program in Solid Tumours, University of Navarra, Centre of Applied Medical Research (CIMA), 55 Pio XII Avenue, Pamplona, 31008 Spain; 2 Centro de Investigacion Biomedica en Red de Cancer (CIBERONC), Madrid, Spain; 3 Imaging Unit and Cancer Imaging Laboratory, University of Navarra, CIMA, Pamplona, Spain; 4 Department of Molecular Biotechnology and Health Sciences, Molecular Biotechnology Centre, University of Torino, Turin, Italy; 5 University of Bordeaux, INSERM, BRIC, Bordeaux, 1312, F‑33000 France; 6 Molecular Therapies Program, University of Navarra, CIMA, Pamplona, Spain; 7 Wellcome - MRC Cambridge Stem Cell Institute (CSCI), Cambridge, UK; 8 Department of Biomedicine, University of Bergen, Bergen, Norway; 9 Bioinformatics Platform, University of Navarra, CIMA, Pamplona, Spain; 10 IdiSNA, Navarra Institute for Health Research, Pamplona, Spain; 11 Department of Pathology, Anatomy and Physiology, University of Navarra, Pamplona, Spain

**Correction** ***Mol Cancer*** **22, 86 (2023)**

10.1186/s12943-023-01788-w

In our publication in Molecular Cancer entitled “The phospholipid transporter PITPNC1 links KRAS to MYC to prevent autophagy in lung and pancreatic cancer” [2023 May 20;22(1):86. doi: 10.1186/s12943-023-01788-w.][1], we regret that the graphical abstract was inadvertently removed from the final version of the manuscript at the production stage. We have provided the corresponding file to be incorporated into the published online version. This correction does not change the scientific conclusions of the article.

The original article has been corrected.
