# Supplementary material for: The phospholipid transporter PITPNC1 links KRAS to MYC to prevent autophagy in lung and pancreatic cancer
Source: Mol Cancer. 2023 May 20;22:86. doi: 10.1186/s12943-023-01788-w (PMC10199551; doi:10.1186/s12943-023-01788-w)
Supplement: Supplementary file 1 — Additional file 1: Suppl. Figure 1. A. PITPNC1 expression levels (log2) in TCGA LUAD patient’s database. Mut EGFR: mutant EGFR, wt: wild type EGFR, N: Normal tissue. Mut vs wt (p=0.014), Mut vs N (p=0.021). B. PITPNC1 expression levels (log2) in TCGA LUAD patient’s database. Mut BRAF: mutant BRAF, wt: wild type BRAF, N: Normal tissue. Mut vs wt (p=0.275), Mut vs N (p=0.336). C. PITPNC1 expression levels (log2) in TCGA LUAD patient’s database with different KRAS point mutations. N: Normal tissue D. Western blot of PITPNC1 and KRAS expression in H1792 cells, expressing a control (GFPsh) or a tet-inducible KRAS shRNA (KRASsh) (activated by 1 μg/ml doxycycline). Twenty μg of protein were loaded per sample. β-TUBULIN was used as loading markes E. PITPNC1 mRNA expression in H2009 and H1792 cells expressing a control (GFPsh) or a tet-inducible KRAS shRNA (KRASsh) (activated by 1 μg/ml doxycycline) (Mann-Whitney or unpaired t-test). F. Western blot of pERK1/2, ERK1/2, pAKT, AKT, p-cJUN, cJUN, pERK5 and ERK5 in A549, H2009 and HPAFII treated with pharmacologic inhibitors: trametinib (MEKi, 0.5 μmol/L), BIX02189 (MEK5i, 10 μmol/L) or GSK2126458 (PI3Ki, 0.1 μmol/L) for 24 h, and SP600125 (JNKi, 10 μmol/L) for 2 h. Twenty μg of protein were loaded per sample. G. PITPNA, PITPNB, PITPNM1, PITPNM2, PITPNM3 mRNA expression in H2126 cells overexpressing a mutant (KRASG12D) or a wild type form (KRAS4B) of KRAS compared to the control (LacZ) (Dunnett’s multiple comparison test). H and I. PITPNA, PITPNB, PITPNM1, PITPNM2, PITPNM3 mRNA expression in H2009 (G) and H1792 (H) cells expressing a control (GFPsh) or an inducible KRAS shRNA (KRASsh) (activated by 1 μg/ml doxycycline) (Mann-Whitney or unpaired t-test). J. Western blot of PITPNC1 and KRAS expression in Kraslox/lox MEFs transduced with different human HA-tagged KRAS mutants (G12C, G12D, G12V, G12R, G12S, G13D and Q61H). K. PITPNC1 expression levels (log2) in TCGA LUAD patient’s database. Mut KRAS: mutant KRAS. Mut KRAS Amp: mutan [file 12943_2023_1788_MOESM1_ESM.docx]

**THE PHOSPHOLIPID TRANSPORTER PITPNC1 LINKS KRAS TO MYC TO PREVENT AUTOPHAGY IN LUNG AND PANCREATIC CANCER**

Rodrigo Entrialgo-Cadierno, Cristina Cueto-Ureña, Connor Welch, Iker Feliu, Irati Macaya, Laura Vera, Xabier Morales, Sandra Vietti Michelina, Pietro Scaparone, Ines Lopez, Elodie Darbo, Oihane Erice, Adrian Vallejo, Haritz Moreno, Ainhoa Goñi-Salaverri, David Lara-Astiaso, Nils Halberg, Ivan Cortes-Dominguez, Elizabeth Guruceaga, Chiara Ambrogio, Fernando Lecanda, Silve Vicent

**SUPPLEMENTARY METHODS**

**Immunoblotting.** Cells were scraped and lysed in buffer containing 1% NP-40, 150 mM NaCl, 50 mM Tris pH 7.4, 1 mM EDTA, 1% glycerol, supplemented with protease inhibitor cocktail (Roche), 25 mM sodium fluoride and 1 mM sodium orthovanadate. Samples were resolved by SDS PAGE and transferred to nitrocellulose membranes (BioRad) and incubated in 5% milk TBS-T for 1h prior to the addition of primary antibodies.

The antibodies used were: PITPNC1 (SAB2701243, 1:1,000, Sigma), KRAS (WH0003845M1, 1:1,000, Sigma), HSP90 (sc-69703, 1:5,000, Santa Cruz Biotech, SBT), β-TUBULIN (T4026, 1:4,000, Sigma), GAPDH (5174, 1:4,000, CST), MYC (5605, 1:1,000, CST), E2F1 (sc-193, 1:500, SBT), CDKN1B/p27 (2552, 1:1,000, CST), LKB1 (3047, 1:1,000, CST), KEAP1 (4678, 1:500, CST), p53 (2524, 1:1,000, CST), STAT3 (4904, 1:500, CST), p-STAT3 (9145, 1:1,000, CST), ERK 1/2 (9102, 1:1,000, CST), p-ERK1/2 (9101, 1:1,000, CST), p-cJUN (3270,1:1,000, CST), cJUN (9165,1:1,000, CST), p-ERK5 (3371, 1:500, CST), ERK5 (3552, 1:500, CST), PLK1 (sc-17783, 1:500, SBT), AURKA (sc- 56881, 1:500; SBT), LC3A/B (4108, 1:1,000, CST), mTOR (2983, 1:1,000, CST), 4EB-P1 (9452, 1:1,000, CST), p4EBP1 T37/46 (9459, 1:1,000, CST), p4EBP1 T70 (9455, 1:1,000, CST), S6 Ribosomal protein (2217, 1:1,000, CST), pS6 Ribosomal protein Ser235/236 (4858, 1:1,000, CST), pS6 Ribosomal protein Ser240/244 (5364, 1:1,000, CST), Caspase3 and cleaved caspase3 (CC3) (9662, 1:1,000, CST). Secondary antibodies used were: Anti-rabbit IgG, (HRP-linked Antibody, 7074, CST) and Anti-mouse IgG (HRP-linked Antibody, NA931V, Amersham).

**Quantitative PCR (qPCR).** Primers sequences used are:

*PITPNC1*: Fw 5’GCGCTACTACAAAGAATCTGAGG3’; Rv 5’GAGCACATGATAGGCTGATGAC3’

*KRAS*: Fw 5’TGGACGAATATGATCCAACAAT3’; Rv 5’TCCCTCATTGCACTGTACTCC3’

*MYC*: Fw 5’GCTGCTTAGACGCTGGATTT3’; Rv 5’ TAACGTTGAGGGGCATCG3’

*p27*: Fw 5’TTGGTGGACCCAAAGACTGAT3’; Rv 5’GTTTTGAGTAGAAGAATCGTCGG3’

*p57*: Fw 5’CTGCACTCGGGGATTTCGG3’; Rv 5’AAATCGGAGATCAGAGGCCC3’

*SESN1*: Fw 5’GCAAATGGATGGGCCGTTAC3’; Rv 5’AGGAGCACTGATGTCTTGCC3’

*SESN2*: Fw 5’GCCACTCAGAGAAGGTCCAC3’; Rv 5’AGGTCATGTAGCGGGTGATG3’

*SESN3*: Fw 5’GACTTTGCCAGACGAGGAGAA3’; Rv 5’CACCAGGGAGAACCCATGATT3’;

*CASTOR1*: Fw 5’GCACATCCTAGAACACCGGG3’; Rv 5’CTGAAGAACTTGCACCGGCT3’;

*PITPNA*: Fw 5’CACGCAGGAGAATGTGCATAAG3’; Rv 5’TGAGCATTGGCTTCGATCT3’;

*PITPNB*: Fw 5’CGAGACTCAGAAAGAACTAGAAA3’; Rv 5’TGACCCTACAGGGGACTCAT3’;

*PITPNM1*: Fw 5’CCAACATGGAGGGGCTGTG3’; Rv 5’TGGGCATCAAAGAACTCTTCCT3’;

*PITPNM2*: Fw 5’GAGTCCTGGAATGCCTACCC3’; Rv 5’GACTGACTGGAACAGCTTGG3’;

*PITPNM3*: Fw 5’CTTCTTCTCCGATGGGCTGG3’; Rv 5’TGATTTTGATGAAGCACTCCTGC3’

*PHLDA2*: Fw 5’CGCCGCGGGCCATAC3’; Rv 5’ACGGGAAGTTCTTCTGCTGC3’

*GJB2*: Fw 5’TCCCGACGCAGAGCAAACC3’; Rv 5’GCTGGTGGAGTGTTTGTTCAC3’

*GPX2*: Fw 5’ACTTCACCCAGCTCAACGA3’; Rv 5’CCTCATTCTGACAGTTCTCCTGAT3’

*BIRC5*: Fw 5’CAAGGACCACCGCATCTCTAC3’; Rv 5’AAGTCTGGCTCGTTCTCAGT3’

*RASSF6*: Fw 5’TAGTGAGCTGGACAGGACCC3’; Rv 5’TGGGGAGTCTGGTTCATCCT3’

*ARK1B10*: Fw 5’GCCTGTAACGTGTTGCAATCCT3’; Rv 5’AGTCACACTTCAGCGAAGAAAG3’

**Cell proliferation assay**. Cell proliferation was evaluated using the CellTiter 96® AQueous Non-Radioactive Cell Proliferation Assay, MTS (Promega). Between 400 and 2,000 cells, depending on the cell line, were seeded in 96 well plates. Experiments were analysed at days 3 and 5 using a spectrophotometer (SpectroStar Nano) and normalized to day 0.

**Clonogenic assay**. 1,000-2,000 cells, depending on the cell line were seeded in 6-well plates (for 14 days). At endpoint, cells were washed with cold PBS twice, fixed with 4% formaldehyde (Panreac) for 15 min at RT, and stained with crystal violet solution (Sigma-Aldrich) (1% crystal violet in H_2_O) for 15 min. Relative growth was quantified by measuring absorbance at 570 nm after crystal violet dissolution 10% with acetic acid.

**Migration assay.** 50,000 cells were placed on the upper layer of a transwell after 24 hours of growth in medium without FBS. In the lower layer, a solution containing 100% FBS (positive control), 10% FBS medium (experimental condition) or medium without FBS (negative control) were used. After 16h, the cells that migrated through the membrane were stained with crystal violet solution (Sigma-Aldrich) (1% crystal violet in H_2_O) and counted.

**3D culture assays**. For 3D culture assays, cold 96-well plates were pre-treated with Matrigel Growth Factor Reduced (MG) (Corning) coating before cell seeding. Cells were resuspended in culture medium (DMEM F12, HEPES 1X, glutamax 1X, primocin 1X, 500 nM TGFβi/A83-01, 50 ng/mL mEGF, mNoggin 100 ng/ml, hFGF10 100 ng/ml, gastrin I 0.01 μM, N-acetylcysteine 1.25 mM, nicotinamide 10 mM, B-27 supplement 1X, R-spondin I-conditioned media 1X, Wnt3a-conditioned media 1X) with 10% MG and 100 μl of the mixture were added to each well. Cells were seeded at 2 x 10^3^ cells per well. Cells were incubated overnight at 37 °C and drugs were added the next day. Proliferation of 3D cultures was measured using CellTiter-Glo® 3D Cell Viability Assay (Promega) according to manufacturer’s instructions.

**Long-term drug combination assays**. 400-2,000 cells, depending on the cell line were seeded in 24-well plates and treated on the next day for 9 days (replacing the media with drug every 3 days). At endpoint, cells were washed with cold PBS twice, fixed with 4% formaldehyde (Panreac) for 15 min at RT, and stained with crystal violet solution (Sigma-Aldrich) (1% crystal violet in H_2_O) for 15 min.

**Cell cycle assay**. Cell cycle analysis was performed using the Click-iT® EdU Flow Cytometry Assay Kit (Invitrogen). Briefly, 300,000 cells were seeded and cultured for 24 h in complete medium. At end point, cells were incubated with 10 µM Edu for 2 h. Next, cells were harvested and washed with 1% of PBS-BSA and fixed in formaldehyde for 15 min at room temperature. Then, cells were washed in 15 PBS-BSA and permeabilized with 1x Click-iT saponin-based reagent for 15 min at room temperature. Next, cells were incubated with the Click-iT reaction cocktail for 30 min at room temperature in the dark. After that, cells were washed with 1x Click-iT saponin-based reagent and incubated with 1 µg/µl of RNase A (Sigma-Aldrich) for 1 h at room temperature. Finally, 7AAD (Invitrogen) was added to the tubes 5 min before cell acquisition in FACSCanto II cytometer (BD Biosciences). Data were analysed using FlowJo® software v10.

**Apoptosis assay**. Apoptosis levels were determined by Annexin V/7AAD assays. Briefly, 400,000 cells were seeded and cultured for 24 h in fully supplemented medium. At end point, cells were harvested and washed with Apoptosis Buffer (0.1 M Hepes (pH 7.4), 1.4 M NaCl, and 25 mM CaCl_2_). Next, cells were centrifuged and resuspended in Apoptosis buffer at a concentration of 1,000,000 cell/ml. 100,000 cells were stained with Annexin V-AlexaFluor-647 (Biolegend) and 7AAD (Invitrogen) for 15 min in the dark. Finally, Apoptosis Buffer was added to the tubes before cell acquisition in FACSCanto II cytometer (BD Biosciences). Data were analysed using FlowJo® software v10.

**Animal work**. For subcutaneous experiments, mice were randomized by age. 2.5 x 10^6^ cells of A549 and 1.5 x 10^6^ cells of PATU8209 infected with lentiviruses carrying specified shRNAs were suspended in 200 μl of 10% FBS-supplemented medium and injected subcutaneously into the two lower flanks of immune-deficient 8 to 12-weeks old *Rag2^-/-^*; *Il2Rγ*^-/-^ mice (Charles River). One-week post-injection, tumour size was measured every three days and tumour volume was calculated using the formula: Volume= π/6 x length x width^2^. Investigator analysis was blinded (samples were coded to a random number).

For the mouse model of lung colonization (tail vein injection) and metastasis (intracardiac injection)^1^, 2 x 10^5^ luciferase expressing A549 cells transduced with pBABE or pBABE-PITPNC1-FLAG suspended in 200 μl of 10% FBS-supplemented medium were injected intravenously. One-week post-injection, every 4 or 7 days, and after administration of 50 µL of Luciferin (15 mg/ml, Promega), bioluminescence was acquired in the ventro-dorsal position using the PhotonIMAGER Optima *in vivo* imaging system (Biospace Lab). At endpoint, macroscopic and microscopic analysis of lung and liver tissues was performed.

Pharmacological inhibition experiments were performed in subcutaneous tumor models derived from the injection of 2 x 10^6^ H358 cells or 4 x 10^6^ MiaPaca2 cells in immune-deficient 8 to 12-weeks old *Rag2^-/-^*; *Il2Rγ*^-/-^ mice (Charles River). Sotorasib (10 mg/kg, once per day) Fedratinib (60 mg/kg, twice per day), or vehicle (10% DMSO, 40% PEG300, 5% Tween-80, 45% saline) were administered for 15 days by oral gavage after the tumors reached an average volume of ~100 mm^3^. Tumor size was measured every 3 days, and tumor volume was calculated using the formula: Volume= π/6 x length x width^2^. Investigator analysis was blinded (samples were coded to a random number).

**Immunofluorescence**. To determine mTOR lysosomal recruitment, 1x10^4^ A549, H2009, or MiaPaca2 cells, or 2x10^4^ HPAFII cells were seeded in 8-well slides (Labteck) covered with 50 µg/mL of collagen type I (BD Biosciences). Afterward, samples were rinsed off with PBS and fixed in 4% PFA at 37°C for 15 minutes. Cells were permeabilized for 10 min with 0.005% Triton X-100, washed with PBS, and blocked nonspecific bindings for one hour at room temperature with 5% BSA. Subsequently samples were incubated overnight at 4°C with the primary antibody anti-mTOR (2983, 7C10, CST) at a dilution of 1:200 in PBS containing 1% BSA. The following day, samples were washed with PBS and incubated for one hour at room temperature with an AlexaFluor 594-conjugated secondary donkey anti-rabbit antibody (1:400, A32754 Thermo Fisher) and AlexaFluor 488-conjugated anti-LAMP1 antibody (328609, clone H4A3, Biolegend) at a 1:50 dilution in PBS with 1% BSA. Nuclei were stained with Hoechst for one hour at room temperature (1:1,000, Thermo Fisher). Finally, Z-stacks of 1024 x 1024 pixels image size were acquired with an oil immersion 63x Plan-Apochromat objective (1.4 NA) on a Zeiss LSM 800 laser-scanning confocal microscope (Carl Zeiss, Jena, Germany). Images were acquired using Zen 2.3 software (Carl Zeiss, Jena, Germany). 3D images of mTOR/LAMP1 stained cells were automatically processed and quantified using the Fiji software^2^. Briefly, raw images were processed as follows: a median filter was applied, and then the background was subtracted using the subtract background tool. Afterwards, the contrast was enhanced by running the CLAHE (Contrast Limited Adaptive Histogram Equalization) plugin, and the background was further reduced by applying the mathematical exponential function (EXP). Z-stacks were binarized using a fixed manual threshold that selects mTOR and LAMP1 clusters. The resulting 3D segmentation masks were analysed using the JACoP^3^ plugin developed for Fiji. This tool implements Mander´s coefficient (MOC), which quantifies the percentage of overlapping pixels above the background between two fluorescent signals. A MOC value of zero indicates no colocalization, while 1 indicates total colocalization. Finally, the analyse particles command was used to automatically identify and measure the size and number of lysosomes per cell using the LAMP1 signal. LAMP1 signal was considered as lysosomes when its diameter ranged from 0.12 to 1.2 microns^4^. Three independent experiments of each cell type were performed, and a total of 20 representative fields containing at least eight cells were acquired and analysed as described above (N~160).

**RNA sequencing analysis**. First, gene expression data was normalized with edgeR^5^ and voom^6^. After quality assessment and outlier detection using R/Bioconductor^7^, a filtering process was performed. Genes with read counts lower than 6 in more than 50% of the samples of all the studied conditions were considered as not expressed in the experiment under study. LIMMA (Linear Models for Microarray Data)^6^ was used to identify the genes with significant differential expression between experimental conditions. Genes were selected as differentially expressed using a B cut-off B > 0. Further functional and clustering analyses and graphical representations were performed using R/Bioconductor^7^.

**Immunohistochemistry.** The following antibodies were used: pH3 (Cell Signaling, 9701; 1:300), CC3 (Cell Signaling, 9661; 1:100) Sections were developed using the DAB+ kit (K346889-2, Dako) and counterstained with Harris hematoxylin.

**SUPPLEMENTARY FIGURES**

**Suppl. Figure 1.** **A**. *PITPNC1* expression levels (log2) in TCGA LUAD patient’s database. Mut EGFR: mutant *EGFR*, wt: wild type *EGFR*, N: Normal tissue. Mut vs wt (p=0.014), Mut vs N (p=0.021). **B**. *PITPNC1* expression levels (log2) in TCGA LUAD patient’s database. Mut BRAF: mutant *BRAF*, wt: wild type *BRAF*, N: Normal tissue. Mut vs wt (p=0.275), Mut vs N (p=0.336). **C**. *PITPNC1* expression levels (log2) in TCGA LUAD patient’s database with different KRAS point mutations. N: Normal tissue **D.** Western blot of PITPNC1 and KRAS expression in H1792 cells, expressing a control (GFPsh) or a tet-inducible *KRAS* shRNA (KRASsh) (activated by 1 µg/ml doxycycline). Twenty μg of protein were loaded per sample. β-TUBULIN were used as loading markers **E.** *PITPNC1* mRNA expression in H2009 and H1792 cells expressing a control (GFPsh) or a tet-inducible *KRAS* shRNA (KRASsh) (activated by 1 µg/ml doxycycline) (Mann-Whitney or unpaired t-test). **F.** Western blot of pERK1/2, ERK1/2, pAKT, AKT, p-cJUN, cJUN, pERK5 and ERK5 in A549, H2009 and HPAFII treated with pharmacologic inhibitors: trametinib (MEKi, 0.5 μmol/L), BIX02189 (MEK5i, 10 μmol/L) or GSK2126458 (PI3Ki, 0.1 μmol/L) for 24 h, and SP600125 (JNKi, 10 μmol/L) for 2 h. Twenty μg of protein were loaded per sample. **G.** *PITPNA, PITPNB, PITPNM1, PITPNM2, PITPNM3* mRNA expression in H2126 cells overexpressing a mutant (KRASG12D) or a wild type form (KRAS4B) of *KRAS* compared to the control (LacZ) (Dunnett’s multiple comparison test). **H and I**. *PITPNA, PITPNB, PITPNM1, PITPNM2, PITPNM3* mRNA expression in H2009 (G) and H1792 (H) cells expressing a control (GFPsh) or an inducible *KRAS* shRNA (KRASsh) (activated by 1 µg/ml doxycycline) (Mann-Whitney or unpaired t-test). **J.** Western blot of PITPNC1 and KRAS expression in *Kras*^lox/lox^ MEFs transduced with different human HA-tagged KRAS mutants (G12C, G12D, G12V, G12R, G12S, G13D and Q61H). **K.** *PITPNC1* expression levels (log2) in TCGA LUAD patient’s database. Mut KRAS: mutant *KRAS*. Mut KRAS Amp: mutant *KRAS* amplification, N: Normal tissue. Mut KRAS vs Mut KRAS Amp (n.s).

**Suppl. Figure 2. A**. *PITPNC1* expression levels (log2) in TCGA LUAD patient’s database in presence of a panel of co-occurrence mutations. **B**. Western blot of LKB1 and PITPNC1 expression in H2009 cells expressing a control (Control) or *LKB1* sgRNAs (*LKB1* sgRNA1 or *LKB1* sgRNA2). Twenty μg of protein were loaded per sample. β-TUBULIN were used as loading markers **C.** Western blot of PITPNC1, p53, Keap1 or Lkb1 expression in KLA and LKR10 cells, expressing a control (Control) or *p53*, *Lkb1* or *Keap1* sgRNAs (p53 sgRNA, Lkb1 sgRNA or Keap sgRNA). Twenty μg of protein were loaded per sample. HSP90 and β-TUBULIN were used as loading markers.

**Suppl. Figure 3. A**. Apoptosis analysis by Annexin V/7AAD labelling in the human LUAD A549, H2009, H1792 and H358 cell lines after *PITPNC1* knockdown with a specific shRNA (sh6 or sh7) compared to the control (GFPsh). (Tukey’s multiple comparison test). **B**. Representative flow cytometry images depicting gating strategy in A. **C**. Representative images of A549- and PATU8902-derived xenografts (GFPsh, PITPNC1 sh6 and PITPNC1 sh7) stained for phospho-histone 3 (pH3) or cleaved caspase 3 (CC3). **D**. Western blot of *PITPNC1* in A549 PITPNC1 OE and H358 PITPNC1 OE cells transfected with a control (pBabe) or *PITPNC1* cDNA. Twenty μg of protein were loaded per sample. β-TUBULIN were used as loading markers **E**. Representative images of clonogenic ability of A549 and H358 PITPNC1 OE cells compared with the control. **F**. Tumour volume (mm^3^) of A549 derived xenografts, (n=8), (Bonferroni’s multiple comparison test). **G**. Representative images of tumours from F. **H**. Tumor weight (g) of tumours in F (n=8) (Mann-Whitney or Unpaired T-test). **I**. Tumour volume (mm^3^) of H358 derived xenografts, (n=8), (Bonferroni’s multiple comparison test). **J**. Representative images of tumours from I. **K**. Tumour weight (g) of tumours in I (n=8) (Mann-Whitney or Unpaired T-test). **L**. Migration assay experiment in *LacZ* and *PITPNC1*-overexpressing A549 and H2009 cell lines. **M**. Representative bioluminescence images of a metastasis assay via intracardiac injection of *LacZ* and *PITPNC1*-overexpressing A549 cells. **N**. *Ex vivo* analysis of bioluminescence in lung, liver and kidney.

**Suppl. Figure 4.** **A**. *PHLDA2, GJB2, GPX2, BIRC5, PITPNC1, RASSF6* and *ARK1B10* mRNA expression in A549 cells expressing a control (GFPsh) or a *PITPNC1* shRNA (sh6 or sh7) (Dunnett´s multiple comparison test). **B**. Box plot comparing early (I-II) and advanced (III-IV) mut *KRAS* LUAD according to the expression of the dPITPNC1 gene signature (GS). **C**. Box plot comparing localized and advanced (locally advanced and metastatic) PDAC based on the expression of the dPITPNC1 gene signature (GS). **D**. Box plot comparing P53 mutant and LKB1 mutant LUAD patients with mut *KRAS* according to the expression of the dPITPNC1 gene signature (GS). **E**. Box plot comparing classical and basal PDAC patients based on the expression of the dPITPNC1 gene signature (GS).

**Suppl. Figure 5.** **A**. Cell cycle analysis by EdU labelling in the human LUAD H1792 and H358 cell lines after *PITPNC1* knockdown with a specific shRNA (sh6 or sh7) compared to the control (GFPsh) (Bonferroni´s multiple comparison test). **B**. Western blot of MYC expression in A549- and PATU8902-xenografts tumours. Twenty μg of protein were loaded per sample. HSP90 and GAPDH were used as loading markers. **C**. Cell cycle analysis by EdU labelling in A549 and Miapaca2 cell lines after *PITPNC1* knockdown with a specific shRNA (sh6 or sh7) compared to the control (GFPsh) (Dunnet´s multiple comparison test). **D**. Western blot of MYC, E2F1 and P27 in A549, H2009, H1792 and Miapaca2 cells, expressing a control (GFPsh) or a shRNA against *PITPNC1* (sh6 or sh7). Twenty μg of protein were loaded per sample. β-TUBULIN was used as loading control. **E**. mRNA analysis of RNAseq data of *E2F1*, *P27* and *P57* in A549 cells expressing a control (GFPsh) or a shRNA against *PITPNC1* (sh6 or sh7). **F** **and G.** QPCR analysis of *P27* (F) and P57 (G) mRNA expression in A549, H2009, H1792 and PATU8902 cells expressing a control (GFPsh) or a *PITPNC1* shRNA (sh6 or sh7) (Dunnett´s multiple comparison test). **H**. Cell proliferation assay in A549 and H2009 cells expressing exogenous LacZ or MYC- and submitted to inhibition of PITPNC1 by specific shRNAs. **I**. Western blot of MYC, PITPNC1 in A549 and H2009 cells expressing exogenous LacZ or MYC- and submitted to inhibition of PITPNC1 by specific shRNAS. Twenty μg of protein were loaded per sample. HSP90 was used as loading control. **J**. RNAseq data of AURKA and PLK1 in A549 expressing a control (GFP) or two PITPNC1 shRNAs

**Suppl. Figure 6. A.** *SESN1*, *SESN2* and *SESN3* expression levels in H2009 cell line were measured by qPCR. Cells were virally infected to express a control (*GFP*sh) or a *PITPCN1* shRNA (sh6 and sh7) (Dunnet´s multiple comparison test). GAPDH was used as housekeeping gene. **B.** mTOR/LAMP1 colocalization analysis by immunofluorescence in H2009 *PITPNC1*-depleted cells. **C.** Quantification of mTOR/LAMP1 Mander´s overlap coefficient (MOC) in H2009 of B (Dunnett’s multiple comparison test). **D.** Western blot of mTOR in A549, H2009 and H1792 cell lines expressing a control (GFP) or two PITPNC1 shRNAs. Twenty μg of protein were loaded per sample and HSP90 was used as loading control. **E**. Lysosomes per cell and average lysosomes size in H2009 of B (Dunn’s multiple comparison test). Lysosomes per cell and average lysosomes size in H2009 of B (Dunn’s multiple comparison test). **F.** Heatmap of autophagy and lysosome biogenesis genes upregulated upon PITPNC1 inhibition in A549 cells (data from RNAseq analysis). **G and H.** Western blot of protein level of LC3-I and LC3-II in A549, H2009 (G) and HPAFII (H) virally infected to express a shRNA control (C) or two *PITPNC1* shRNAs (sh6 and sh7) and treated with or without hidroxychloroquine (CQ) (60 μM) for 6 h. Twenty μg of protein were loaded per sample and HSP90 was used as loading control. **I.** Western blot of mTOR signalling pathway (mTOR, 4EBP1 and S6K) in A549, H2009 and H1792 cell lines in which *PITPNC1* was inhibited with a specific shRNA (sh6 and sh7). Twenty μg of protein were loaded per sample and HSP90 was used as loading control.

**Suppl. Figure 7. A.** mTOR/LAMP1 colocalization analysis by immunofluorescence in A549 *MYC*-depleted cells. **B.** Quantification of mTOR/LAMP1 Mander´s overlap coefficient (MOC) in A549 of A (Dunnett’s multiple comparison test). **C.** mTOR/LAMP1 colocalization analysis by immunofluorescence in A549 *MYC*-depleted cells. **D.** Quantification of mTOR/LAMP1 Mander´s overlap coefficient (MOC) in MiaPaca2 of C (unpaired t-test). **E.** Lysosomes per cell and average lysosomes size in A549 and MiaPaca of A and C respectively (Dunn’s multiple comparison test and unpaired t-test respectively). **F**. Lysosomes per cell and average lysosomes size in A549 and MiaPaca of A and C respectively (Dunn’s multiple comparison test and unpaired t-test respectively). **G.** *SESN1*, *SESN2* and *SESN3* expression levels in A549 cell line were measured by qPCR. Cells were virally infected to express a control (*GFP*sh) or a *Myc* shRNA (sh42 and sh89) (Dunnet´s multiple comparison test). GAPDH was used as housekeeping gene. **H.** *SESN1*, *SESN2* and *SESN3* expression levels in Miapaca2 cell line were measured by qPCR. Cells were virally infected to express a control (*GFP*sh) or a *Myc* shRNA (sh42 and sh89) (Dunnet´s multiple comparison test). GAPDH was used as housekeeping gene.

**Suppl. Figure 8. A.** Connectivity Map (CMap) analysis for dKRAS GS H358 transcriptomics. Perturbagen classes with mean connectivity scores >90% are displayed. Each dot represents an individual drug included in the specific class.. **B**. Representative image of crystal violet stained plates for drug combination experiment in G12C cell lines. **C**. Synergistic score (Bliss score) heatmaps of H1792, H2030, H358, H23 and MiaPaca2 treated for 5 days as indicated **D**. Representative image of 3D proliferation assay in H358 and H1792 cell lines treated with DMSO (Ctrl) Soto (60 nM) Fedra (1 μM) or both (Combo) for 5 days. **E**. Representative image of crystal violet stained plates for drug combination experiment in Sotorasib-resistant (KR) G12C cell lines H358 and H23.

**Suppl. Figure 9. A and B.** Representative images of H358- and Miapaca2-derived xenografts stained for phospho-histone 3 (pH3) or cleaved caspase 3 (CC3). **C and D**. Mouse weight change upon different treatments. S= start of the experiment; E= end of the experiment, (Mann-Whitney or unpaired t-test).

**SUPPLEMENTARY REFERENCES**

1. Luis-Ravelo, D. *et al.* RHOB influences lung adenocarcinoma metastasis and resistance in a host-sensitive manner. *Mol Oncol* **8**, 196–206 (2014).

2. Schindelin, J. *et al.* Fiji: an open-source platform for biological-image analysis. *Nat Methods* **9**, 676–682 (2012).

3. Bolte, S. & Cordelières, F. P. A guided tour into subcellular colocalization analysis in light microscopy. *J Microsc* **224**, 213–232 (2006).

4. de Araujo, M. E. G., Liebscher, G., Hess, M. W. & Huber, L. A. Lysosomal size matters. *Traffic* **21**, 60–75 (2020).

5. Robinson, M. D., McCarthy, D. J. & Smyth, G. K. edgeR: a Bioconductor package for differential expression analysis of digital gene expression data. *Bioinformatics* **26**, 139–140 (2010).

6. Ritchie, M. E. *et al.* limma powers differential expression analyses for RNA-sequencing and microarray studies. *Nucleic Acids Res* **43**, e47 (2015).

7. Gentleman, R. C. *et al.* Bioconductor: open software development for computational biology and bioinformatics. *Genome Biol* **5**, R80 (2004).
